# Protein catalyzed capture agents with tailored performance for *in vitro* and *in vivo* applications

**DOI:** 10.1002/bip.22934

**Published:** 2017-03-25

**Authors:** Matthew B. Coppock, Candice R. Warner, Brandi Dorsey, Joshua A. Orlicki, Deborah A. Sarkes, Bert T. Lai, Suresh M. Pitram, Rosemary D. Rohde, Jacquie Malette, Jeré A. Wilson, Paul Kearney, Kenneth C. Fang, Scott M. Law, Sherri L. Candelario, Blake Farrow, Amethist S. Finch, Heather D. Agnew, James R. Heath, Dimitra N. Stratis‐Cullum

**Affiliations:** ^1^ Sensors and Electron Devices Directorate U.S. Army Research Laboratory Adelphi MD 20783; ^2^ Excet, Springfield VA 22151 supporting USA Edgewood Chemical Biological Center Aberdeen Proving Ground MD 21010; ^3^ Federal Staffing Resources, Annapolis, MD supporting U.S. Army Research Laboratory Adelphi MD 20783; ^4^ Weapons and Materials Research Directorate U.S. Army Research Laboratory Aberdeen Proving Ground MD 21005; ^5^ Indi Molecular 6162 Bristol Parkway Culver City CA 90230; ^6^ Integrated Diagnostics Seattle WA 98109; ^7^ Division of Chemistry and Chemical Engineering California Institute of Technology 1200 East California Boulevard Pasadena CA 91125

**Keywords:** biological stability, protective antigen, protein catalyzed capture agent, synthetic antibody, thermal stability, peptide, vascular endothelial growth factor

## Abstract

We report on peptide‐based ligands matured through the protein catalyzed capture (PCC) agent method to tailor molecular binders for *in vitro* sensing/diagnostics and *in vivo* pharmacokinetics parameters. A vascular endothelial growth factor (VEGF) binding peptide and a peptide against the protective antigen (PA) protein of *Bacillus anthracis* discovered through phage and bacterial display panning technologies, respectively, were modified with click handles and subjected to iterative *in situ* click chemistry screens using synthetic peptide libraries. Each azide‐alkyne cycloaddition iteration, promoted by the respective target proteins, yielded improvements in metrics for the application of interest. The anti‐VEGF PCC was explored as a stable *in vivo* imaging probe. It exhibited excellent stability against proteases and a mean elimination *in vivo* half‐life (*T*_1/2_) of 36 min. Intraperitoneal injection of the reagent results in slow clearance from the peritoneal cavity and kidney retention at extended times, while intravenous injection translates to rapid renal clearance. The ligand competed with the commercial antibody for binding to VEGF *in vivo*. The anti‐PA ligand was developed for detection assays that perform in demanding physical environments. The matured anti‐PA PCC exhibited no solution aggregation, no fragmentation when heated to 100°C, and  > 81% binding activity for PA after heating at 90°C for 1 h. We discuss the potential of the PCC agent screening process for the discovery and enrichment of next generation antibody alternatives.

## 1 Introduction

Monoclonal antibodies (mAbs) are the current standard as biological recognition elements for *in vitro* and *in vivo* applications. While the reputation of mAbs for high affinity and selectivity is often overstated,^1^ they do consistently perform better than most reported alternative capture agent technologies. However, thermal and biological instabilities, batch‐to‐batch variability, and molecular size can limit the effectiveness of mAbs. For example, antibodies often lack the stability to serve as capture agents for environmentally demanding applications.^2^ As a second example, the large size of mAbs can yield attractive pharmacokinetic (PK) properties for certain therapeutic tasks. However, their large size can also prevent the rapid *in vivo* clearance often required for *in vivo* imaging tasks.^3^, ^4^ Some of these drawbacks are being actively addressed,^5^ including the work by McConnell et al. to produce stabilized IgG frameworks that can maintain >60% binding activity after 1 h at 90°C.^6^

Various oligonucleotides,^7^ small biologics,^8^, ^9^, ^10^, ^11^ or peptide capture agents that are developed through screening methods may be engineered for such demanding tasks. For example, several small biologics have been shown to exhibit high melting onsets in buffered solution. These include Adhirons (101°C),^12^ Affibodies (>90°C),^13^ and DARPins (up to 96°C),^11^ although the binding activity following such thermal treatments has not been reported. The common definition of thermal stability (*T*_m_), as applied to these alternate scaffolds, does not capture the full range of relevant properties, since a high melting point reagent can lose significant binding activity after prolonged exposure to high heat. A relevant example is a single domain antibody‐maltose fusion protein.^14^ Small mAb biologics can exhibit improved tissue and solid tumor penetration relative to mAbs, but can also exhibit short serum half‐lives due to enzymatic degradation and rapid renal clearance.^15^

Peptide‐based ligands are straightforward enough to develop using cellular display methods,^16^, ^17^ but typically exhibit target selectivity and affinity properties that are inferior to mAbs. However, such peptide structures are—like small molecules—not susceptible to misfolding, and are amenable to strategic molecular alterations. Methods for improving the performance of such artificial antibodies have advanced in recent years. Herein we explore protein catalyzed capture (PCC) agent technology^18^ as a method for iteratively improving the properties of peptide ligands originally discovered through alternative approaches.^19^

For the PCC method used here, the original ligand is chemically modified to present an azide (or alkyne) click handle and a biotin assay handle. This modified ligand is called the anchor. The anchor is then incubated with a large one‐bead‐one‐compound (OBOC) peptide library designed to present the complementary alkyne (or azide) click handle. The azide‐alkyne cycloaddition reaction is commonly promoted by a Cu(I) catalyst.^20^, ^21^ For an *in situ* click screen, no Cu(I) catalyst is used. Instead, the protein target, itself, provides an exquisitely selective scaffold by promoting the click reaction between the target‐bound anchor and those few library elements that will bind to the target in just the right geometry relative to the anchor.^[18a]^ 18 Unlike most screening methods, the PCC approach does not screen for the binding of library elements to the target. Instead, the screen is designed to identify the *in situ* click reaction product, which are those beads that contain covalently coupled anchor. The PCC process can be used to develop linear, branched, and/or cyclic peptide architectures.

In the present study, we explore the tunability of 2 different PCCs for specific *in vitro* and *in vivo* applications. Each PCC anchor was developed from a peptide ligand identified using bacterial or phage display screening, and subsequently tailored for the targeted application through the use of *in situ* click screens. We describe the development, as well as the resulting *in vivo* performance and biological stability metrics of an anti‐vascular endothelial growth factor (VEGF) PCC. Additionally, we provide an in‐depth study of the stability and stability limits of a recently described anti‐protective antigen (PA) PCC under physically demanding conditions for *in vitro* detection. We illustrate that the PCC approach can be utilized to mature ligands for specific *in vivo* and *in vitro* applications for which mAbs may not be well suited.

## 2 Materials and methods

### 2.1 Materials

PA stock, recombinant from *E. coli*, NR‐3780 was obtained through the NIH Biodefense and Emerging Infections Research Resources Repository, NIAID, NIH. The Anthrax PA measured by circular dichroism was obtained from List Labs (Lot: 17115A6B). Mouse anti‐*Bacillus anthracis* PA monoclonal antibody was purchased from US Biological Life Sciences, and all surface plasmon resonance (SPR) chips were purchased from GE Healthcare/Biacore Life Sciences. Recombinant human VEGF (VEGF165) and VEGFR2 proteins and were purchased from R&D Systems, and Bevacizumab (Avastin^®^; BVZ) was purchased from Besse Medical. Bevacizumab Fab (BVZ Fab) was generated by digestion with papain using the Pierce™ Fab Micro Preparation Kit. All chemicals for peptide synthesis were acquired from AAPPTec. All additional chemicals were purchased from Sigma‐Aldrich and BDH Chemicals.

### 2.2 Human plasma stability

Human plasma was pre‐warmed at 37°C for 5 min, followed by addition of 5 µ*M* **Anchor_V_** or **Bi‐L_V_** with a final DMSO concentration of 0.5% (v/v). The incubation was performed in a 37°C water bath for 0, 15, 30, 45, and 60 min. At each time point, an aliquot of the incubation mixture was transferred to acetonitrile. Samples were then mixed and centrifuged. Supernatants were analyzed by HPLC‐MS/MS analysis (Agilent 1200 HPLC and a 5500 Qtrap Applied Biosystems Mass Spectrometer) using selected reaction monitoring. The HPLC system consisted of a binary LC pump with an autosampler, a C‐18 column (Gemini C18, 2.1 × 50 mm, 5 micron; Phenomenex), and a gradient (Table S6, Supporting Information). Peak areas corresponding to the test compound were recorded. The compound remaining (%) was calculated by comparing the peak area at each time point to time zero.

### 2.3 Mouse liver microsome stability

The **Bi‐L_V_** was pre‐incubated with pooled mouse liver microsomes (male CD‐1, 0.3 mg/mL) in phosphate buffer (pH 7.4) for 5 min in a 37°C shaking water bath. The concentration of test compound was 1 µ*M* with 0.01% DMSO, 0.25% acetonitrile, and 0.25% methanol. The reaction was initiated by adding a NADPH‐generating system (1.3 m*M* NADP, 3.3 m*M* G6P, and 0.4 U/mL G6PDHase) and incubated for 0, 15, 30, 45, and 60 min. The reaction was stopped by transferring the incubation mixture to an equal volume of acetonitrile/methanol (1/1, v/v). Samples were then mixed and centrifuged. Supernatants were used for HPLC‐MS/MS analysis with selected reaction monitoring. Peak areas corresponding to the test compound were recorded and the compound remaining (%) was calculated by comparing the peak area at each time point to time zero.

### 2.4 *In vivo* pharmacokinetics (PK)

#### 2.4.1 Sample collection from mice (parallel sampling)

Experiments were performed as specified in Supporting Information, Tables S2–S4. Briefly, plasma concentrations and pharmacokinetics of anti‐VEGF PCC agents were studied in groups of 24 mice after IV or IP administration at a single dose rate of 1 or 5 mg/kg body weight, respectively. Animals were sedated under general inhalant anesthesia (3% isoflurane) for blood collection by cardiac puncture. Each mouse was subject to one blood draw. For IV groups, blood was drawn from each of three animals at the following time points: 3, 10, 30, 60, 120, 240, 360, 1,440 min. For IP groups, blood was drawn from each of three animals at the following time points: 10, 30, 60, 120, 240, 360, 480, 1,440 min. Blood aliquots (300–400 µL) were collected in tubes coated with lithium heparin, mixed gently, then kept on ice and centrifuged at 2,500*g* for 15 min at 4°C, within 1 h of collection. The plasma was then harvested and kept frozen at −20°C until further processing.

#### 2.4.2 Sample analysis

The LC‐MS/MS system for analysis was comprised of an Agilent 1200 high‐performance liquid chromatography (HPLC) and a 5500 Qtrap mass spectrometer (Applied Biosystems by Life Technologies, Carlsbad, CA). Concentrations in mouse plasma were determined using a nonvalidated LC‐MS/MS assay with Angiotensin I as the internal standard following solid phase extraction. Individual calibration and QC standards were prepared from the respective intermediate stock solutions by addition to naïve mouse plasma. The target working range of the calibration curve was 50–5,000 ng/mL in plasma, and the target concentrations of the QC standards were 150–3,750 ng/mL in plasma.

Pharmacokinetic analysis was performed using WinNonlin Professional Edition (Pharsight Corporation, Version 5.2). Nominal dose levels and sample collection times were used. Ratios and descriptive statistics were calculated using WinNonlin or Microsoft Excel (Version 11.0). Values below the lower limit of quantitation (BLQ) were treated as zero for descriptive statistics and were excluded from pharmacokinetic analysis. Predose concentrations for IV dose groups were excluded to allow for back extrapolation to concentration at time 0.

### 2.5 MicroPET imaging of non‐tumor‐bearing mice

Female immunocompromised NU/J mice (6–7 weeks old) were obtained from Jackson Laboratory. Mice were weighed and subjected to general clinical observations. Experiments were performed according to Institutional Animal Care and Use Committee (IACUC) guidelines set forth by MPI Research.

The 1,4,7,10‐tetraazacyclododecane‐*N,N′,N″,N″′*‐tetraacetic acid (DOTA)‐labeled **Tri‐L_V_** was radiolabeled by chelation with ^64^Cu. Radiolabeling was performed for 1 h with 47–84% radiochemical yields. Imaging was performed on the non‐tumor‐bearing mice after injection of ^64^Cu‐DOTA‐labeled **Tri‐L_V_** (MALDI‐MS (m/z): calcd. for C_224_H_331_N_61_O_66_S_3_ (M^+^) 5027.4; found (M + H) 5030.7.) in a microPET (Focus 120, Siemens Medical Solutions), followed by CT. Mice (*n* = 3) were administered 200 µCi (65 µg) of ^64^Cu‐DOTA‐**Tri‐L_V_** via IV or IP injection. A dynamic imaging scan was conducted from 0 to 2 h postdose, and static imaging scans were conducted for 15 or 30 min at 4 and 20 h postdose, respectively, for all animals. The data reconstructed from PET and CT were visualized and coregistered using VivoQuant™ software (inviCRO, LLC).

### 2.6 MicroPET‐CT imaging of mouse tumor model

VEGF‐positive human HT‐29 colon adenocarcinoma tumor cells [5 × 10^6^ cells prepared in 100 μL of serum‐free culture medium/Matrigel (1:1)] were inoculated on the left rear flanks of female immunocompromised NU/J mice to result in subcutaneous xenografts. Imaging was performed on the xenografted mice after 20 h post injection of ^64^Cu‐DOTA‐**Tri‐L_V_** in a microPET, followed by CT. Mice were administered 200 μCi (65 μg) of ^64^Cu‐DOTA‐**Tri‐L_V_** via IP injection. For blocking experiments, animals received an IV bolus injection of 100 μL unlabeled BVZ (1 mg) at 48 h (±2 h) before the IP administration of ^64^Cu‐DOTA‐**Tri‐L_V_**, since BVZ and the triligand would target the same human VEGF epitope, leading to the decreased accumulation of ^64^Cu‐DOTA‐**Tri‐L_V_** in the tumor. BVZ was prepared fresh by diluting stock in vehicle (0.9% NaCl for Injection, USP) prior to dosing.

### 2.7 HPLC and mass spectrometry

Five separate 100 µL aliquots of a 1 mg/mL sample of **Bi‐L_PA_** in PBS were heated at 25°C, 70°C, 80°C, 90°C, and 100°C for 60 min, respectively. After heating, the samples were immediately put on ice. Three microliter of each sample was injected into an Agilent 1200 series analytical HPLC and through an Agilent Eclipse XBD‐C18 column, with an elution gradient of 20–60% acetonitrile in 0.1% trifluoroacetic acid in water over 35 min. The samples were monitored at 280 nm.

The mass measurement of each heated sample was performed on a Shimadzu Axima‐CFR MALDI‐TOF in linear mode with a N_2_ laser. The laser power was set at 110–120 and the sample was mixed with a matrix of 10 mg/mL 2,5‐dihydroxybenzoic acid (DHB).

### 2.8 Thermofluor assay

Thermal aggregation studies were performed using the ProteoStat Thermal Shift Stability Assay (Enzo Life Sciences). 1 and 15.5 mg/mL concentrations of **Bi‐L_PA_** were studied in 5% DMSO in the assay buffer provided with the kit. Anti‐*Bacillus anthracis* PA mAb was diluted to a concentration of 0.2 mg/mL in assay buffer. The fluorescence of each sample was read using the LightCycler 480 system (Roche) at 498 nm excitation and 610 nm emission, while heating the samples from 25 to 99°C at 0.2°C/min.

### 2.9 Enzyme linked immunosorbent assay (ELISA)

Two 650 µL (25 µ*M*) solutions of **Bi‐L_PA_** were prepared in PBS. One solution was heated for 1 h at 70°C and allowed to equilibrate to room temperature. Eleven serial dilutions from 25 to 0.012 µ*M* were prepared with each **Bi‐L_PA_** solution. Assays were performed using the previously described detailed protocol^19^ ^[19b]^ and run in triplicate.

### 2.10 Surface plasmon resonance thermo activity assay

RU (30,000) of recombinant PA was amine‐coupled to a Biacore T200 series S sensor chip CM5. **Bi‐L_PA_** was diluted to 1 mg/mL in PBS, aliquoted in 100 µL volumes, and heated to 90°C for 15, 30, 45, and 60 min. After each time point, samples were pulled and immediately put on ice. One sample was left unheated. A calibration curve of unheated sample was created by injecting 25, 50, 100, 150, 200, 250, and 300 µ*M* over the PA surface. One control sample was run at 175 µ*M* every 25 injection cycles to ensure the sensor surface was not degraded over time. Heated samples for each time point (0, 15, 30, 45, and 60 min) were diluted 1:1.5 in PBS and plotted on the calibration curve using BiaEvaluation software to determine active concentration. All samples were evaluated in a Biacore T200 SPR instrument. Activity was plotted relative to the control sample with standard deviation.

## 3 Results and discussion

The PCC method was used to improve an anti‐VEGF peptide as a stable *in vivo* imaging probe and an anti‐PA peptide as an *in vitro* detection reagent suitable for demanding physical environments. We first characterized the *in vitro* properties of the anti‐VEGF peptide and then tracked the stepwise improvements in affinity, selectivity, and stability after each *in situ* click screen. We then focused our efforts on studying the *in vivo* properties of the biligand and triligand. The synthetic flexibility of the PCC method permitted us to attach site‐specific labels to ligands for the *in vitro* assays, or radiolabels, for the *in vivo* assays described below.

### 3.1 Anti‐VEGF PCCs: (*In vitro*) affinity, selectivity, and stability maturation

**Figure 1:**
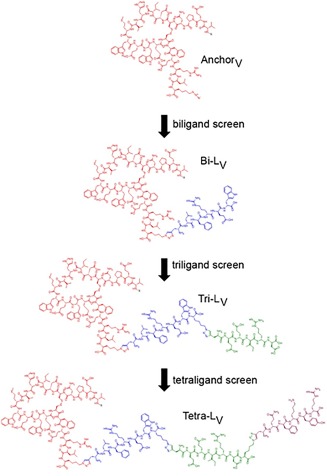
Maturation of an anti‐VEGF PCC agent. X = detection label

**Figure 2:**
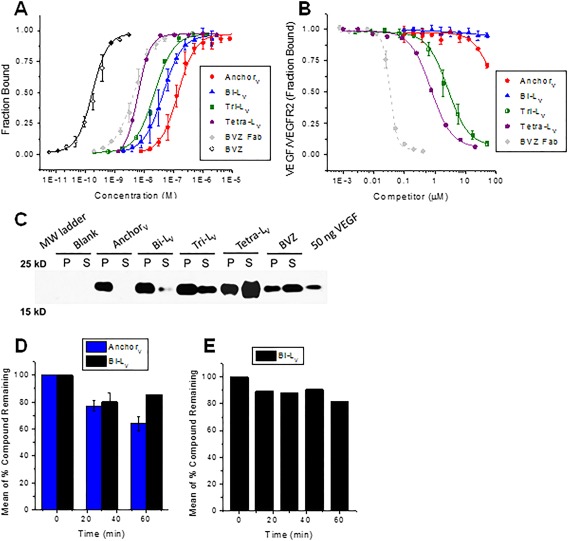
*In vitro* assays of anti‐VEGF PCCs. (A) Relative affinities of PCCs compared to Bevacizumab (BVZ) and Bevacizumab Fab (BVZ Fab). (B) Inhibition of VEGF binding to VEGFR2 in the presence of PCCs and BVZ Fab. (C) Immunoprecipitation of VEGF from PBS (P) and 25% human serum (S) for PCCs as compared to BVZ. (D) Stability of **Anchor_V_** and **Bi‐L_V_** in human plasma at 37°C. (E) Stability of **Bi‐L_V_** in mouse liver microsomes at 37°C

We developed an anti‐VEGF PCC as a positron emission tomography (PET) probe for *in vivo* imaging. We began by maturing a phage display‐derived, literature‐based anti‐VEGF cyclic peptide ligand^22^ into a biligand using an *in situ* click screen (see Supporting Information for experimental details).^18^ ^[18c,^ ^19a]^ **Anchor_V_** (X‐VEPNCDIHVMWEWECFERL‐Az4) was obtained by modifying the cyclic peptide with a biotin‐PEG_3_ label (= X, Figure 1) and an azide‐containing amino acid (Az4 = l‐azidolysine). A disulfide bridge was formed between the two cysteine thiols to cyclize the underlined region of **Anchor_V_**. **Anchor_V_** exhibited an EC_50_ = 140 ± 21 n*M*, which represents good binding to VEGF, but an improved affinity would be needed to be competitive with either the anti‐VEGF monoclonal antibody BVZ (EC_50_ = 0.17 ± 0.02 n*M*) or the monovalent BVZ Fab (EC_50_ = 4.6 ± 0.3 n*M*) (Figure 2A). Inhibition assays further showed that **Anchor_V_** weakly binds to a common epitope that is implicated in the binding of VEGF to its cognate receptor, VEGFR2 (KDR) (Figure 2B). **Anchor_V_** exhibited insufficient selectivity to capture VEGF from (diluted to 25%) serum samples in an immunoprecipitation experiment (Figure 2C).

**Scheme 1:**
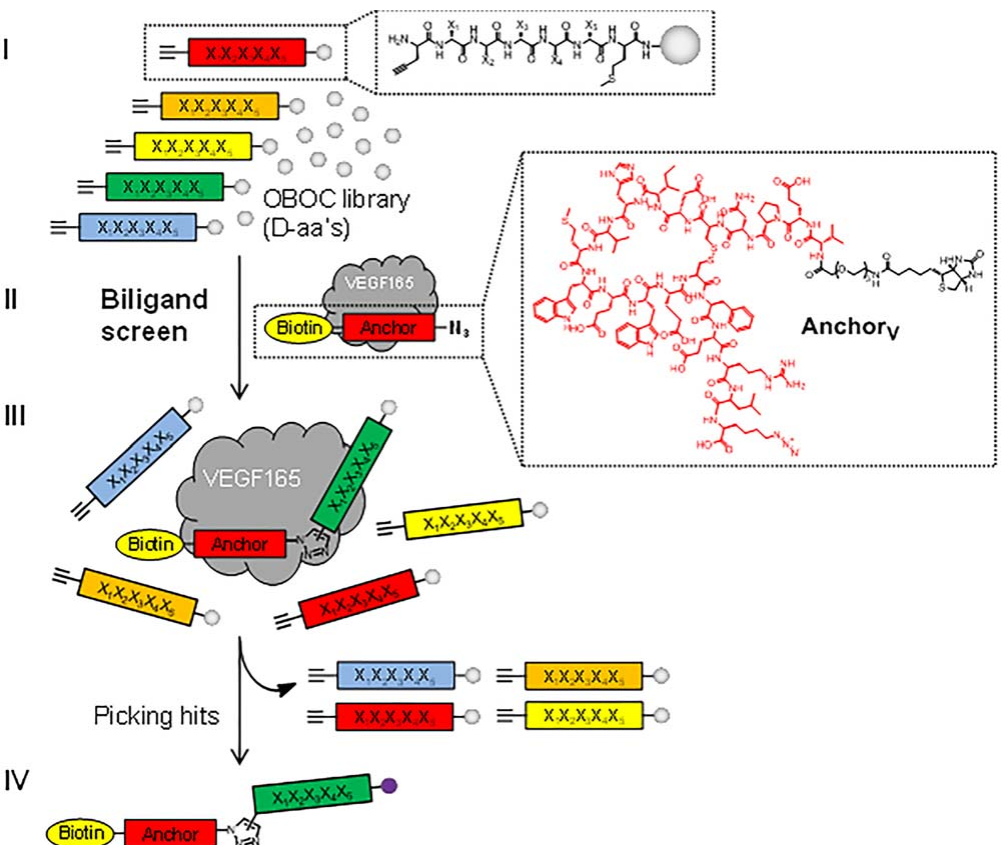
*In situ* click screening of VEGF. A randomized pentapeptide library (X = 18 d‐amino acids) is synthesized on TentaGel resin. d‐propargylglycine is fixed at the N‐terminus, and d‐methionine is fixed at the C‐terminus (I). This library is incubated with VEGF165 and Biotin‐labeled **Anchor_V_** in a biligand screen (II). Target binding is detected by anti‐VEGF165 antibody followed by an alkaline phosphatase (AP)‐conjugated secondary antibody. The hit beads are washed, stripped, and reprobed with AP‐conjugated streptavidin to detect products of the target‐catalyzed *in situ* click chemistry (III‐IV). Methionine‐specific CNBr cleavage and sequencing by MALDI‐TOF/TOF yield the sequences of biligand candidates. The biligand candidates are synthesized on a larger scale and assayed to assess *in vitro* performance (affinity, selectivity, stability, etc.). Repeating the process once yields triligands and twice results in tetraligands

**Anchor_V_** was matured by *in situ* click screening against VEGF165 and a one‐bead–one‐compound (OBOC) D‐peptide library terminated with the complementary alkyne. Scheme 1 illustrates the general strategy for using the VEGF target to template the formation of biligands by combining a library of synthetic peptides with **Anchor_V_**. Several anti‐VEGF biligand PCCs with improved binding towards VEGF165 (Figures S3–S4, Supporting Information) were identified by this approach. Although there was no significant difference in affinity between the biligands, **Bi‐Lv** showed more significant capture of VEGF in immunoprecipitation studies from 25% human serum. **Bi‐L_V_** (X‐VEPNCDIHVMWEWECFERL‐Tz4‐lfrew) exhibited a 4.5‐fold improvement in EC_50_ relative to **Anchor_V_** (Figure 2A), but did not compete as effectively as **Anchor_V_** for the VEGFR2 binding site (Figure 2B). **Bi‐L_V_** could be used to immunoprecipitate VEGF from 25% human serum (Figure 2C) and is therefore more selective than **Anchor_V_**.

The biological stabilities of **Anchor_V_** and **Bi‐L_V_** in human plasma were also compared. Bi‐L showed an improved stability, with > 80% of the peptide remaining after incubation in human plasma for 60 min at 37°C (Figure 2D). **Bi‐L_V_** also showed a high resistance to metabolic degradation by mouse liver microsomes (Figure 2E). This excellent biological stability almost certainly results from the introduction of proteolysis resistant d‐amino acids, and so, such amino acids were used for further iterative improvements of this PCC.^23^, ^24^

The improved *in vitro* performance metrics of **Bi‐L_V_** prompted us to study this PCC *in vivo* (see next section) and to further mature the PCC via *in situ* click screening into a consensus triligand (**Tri‐L_V_**, X‐VEPNCDIHVMWEWECFERL‐Tz4‐lfrew‐Tz4‐eeird) (Supporting Information, Figures S7–S9) and tetraligand (**Tetra‐L_V_**, X‐VEPNCDIHVMWEWECFERL‐Tz4‐lfrew‐Tz4‐eeird‐Tz4‐qfkyr) (Supporting Information, Figures S12 and S13). Each *in situ* click screen iteration yielded improvements in both binding affinity and selectivity, with **Tetra‐L_V_** (EC_50_ = 6.1 ± 0.2 n*M*) exhibiting performance comparable to the BVZ Fab (Figures 2A–2C). Both **Tri‐L_V_** and **Tetra‐L_V_** exhibited immunoprecipitation of VEGF from 25% human serum that was similarly comparable to that of BVZ. The inhibition of VEGF binding to VEGFR2 was also improved for both **Tri‐L_V_** (IC_50_ = 2.6 ± 0.5 n*M*) and **Tetra‐L_V_** (IC_50_ = 0.74 ± 0.05 n*M*). **Tetra‐L_V_** provides a demonstration of what can be achieved using the iterative *in situ* click approach. However, as a synthetic peptide, it is a bit unwieldy for *in vivo* applications. Thus, we pushed **Bi‐L_V_** and **Tri‐L_V_** forward for those studies.

### 3.2 Anti‐VEGF PCC: (*In vivo*) PK analysis and targeting

The ability to chemically tailor the size and stability of PCCs can be useful for *in vivo* applications such as PET molecular imaging. We radiolabeled the anti‐VEGF PCCs **Bi‐L_V_** and **Tri‐L_V_** for studying, *in vivo* in mice, their pharmacokinetics (PK) for different routes of administration. We then showed that radiolabeled **Tri‐L_V_** finds its target *in vivo* by PET imaging.

PK describes the time course of the level of an administered compound in the body. Smaller molecules generally exhibit rapid (<1 h) body clearance and improved tissue penetration, while reagents >∼70 kDa often circulate for much longer times.

**Figure 3:**
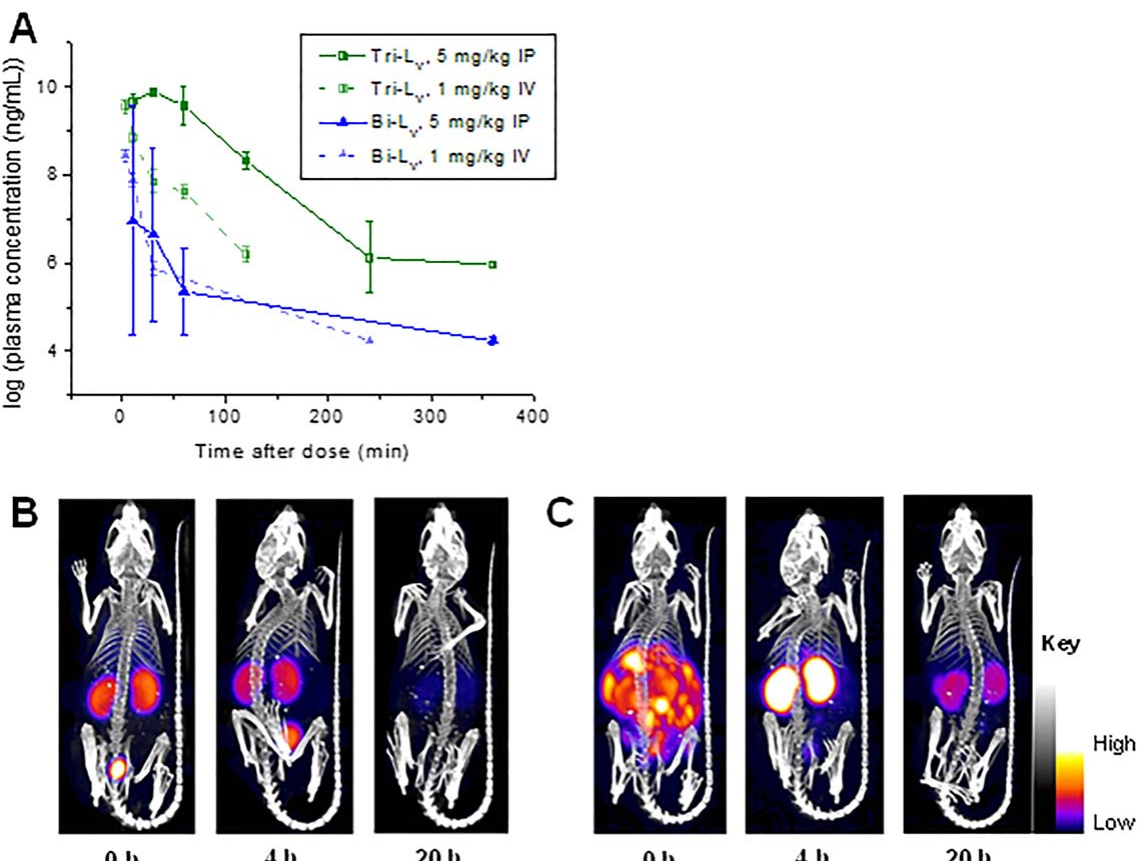
*In vivo* assays of anti‐VEGF PCCs. (A) Pharmacokinetics profiles of **Bi‐L_V_** and **Tri‐L_V_** following a single IV or IP dose in mice. (B) *In vivo* microPET‐CT images (coronal slices) of nude mice demonstrating renal clearance of 64Cu‐DOTA‐labeled **Tri‐L_V_** administered IV. (C) *In vivo* microPET‐CT images of 64Cu‐DOTA‐labeled **Tri‐L_V_** for the same mice administered IP

The PK of **Bi‐L_V_** and **Tri‐L_V_** was performed following intravenous (IV) and intraperitoneal (IP) dosing to mice. Concentration‐time profiles for each compound and route of administration are shown in Figure 3A, and the calculated PK parameters are summarized in the Supporting Information (Table S5). The clearance of the PCCs was similar to other peptides.^24^ Following IV administration of **Bi‐L_V_**, the mean elimination half‐life (*T*_1/2_) is 7 min, which represents a rapid *in vivo* clearance of **Bi‐L_V_**. **Tri‐L_V_**, as an iterated PCC with increased molecular size, allowed us to extend the *in vivo* clearance to *T*_1/2_ = 36 min (by IV). This, and the other PK values (Table S5), imply that **Tri‐L_V_** provides more favorable PK characteristics for *in vivo* PET imaging,^4^ where relatively short circulation times with rapid target visualization are desirable.

**Tri‐L_V_** was prepared as an N‐terminal DOTA conjugate and then radiolabeled (see Methods) with ^64^Cu by chelation. The *in vivo* biodistribution of radiolabeled **Tri‐L_V_** was characterized using PET imaging following either IV or IP injection into healthy mice. PET scans displayed *in vivo* kinetics that are dependent on the route of administration. IV injection resulted in rapid renal clearance (Figure 3B). IP injection resulted in slow clearance from the peritoneal cavity and kidney retention at extended times (Figure 3C). In either case, the majority of the PCC cleared to the kidneys and bladder by 4 h postinjection. Because of the relatively short half‐life of **Tri‐L_V_**, an IP route of administration was prioritized for further PET imaging studies.

A mouse model of human HT‐29 colorectal carcinoma was used to study whether the radiolabeled **Tri‐L_V_** is capable of binding to VEGF in a tumor *in vivo*. This model expresses VEGF and is characterized by VEGF‐dependent radial tumor growth.^25^ Mice bearing HT‐29 xenograft tumors in their rear flanks were imaged by PET‐CT with radiolabeled **Tri‐L_V_**‐administered IP (Figure S14A, Supporting Information). To determine specificity for human VEGF, unlabeled excess BVZ (1 mg) was preinjected prior to imaging to block the target *in vivo*. Pretreatment with BVZ was found to reduce tumor accumulation of radiolabeled **Tri‐L_V_** at 20 h and suggests binding to a shared VEGF epitope *in vivo* (Figure S14B). In the tumor was observed a 32% blockage (*P* = 0.004) of radiolabeled **Tri‐L_V_** uptake by BVZ.

### 3.3 Anti‐VEGF PCC: Synthesis on solid‐phase

Once identified, each PCC is synthesized on a larger scale on acid‐cleavable polystyrene resin. As a consequence of the *in situ* click screen, a triazole (Tz) connects pairs of peptide ligands via their terminal side chains of Az4 and d‐propargylglycine. A commercial peptide synthesizer employing standard Fmoc/HBTU chemistry was used to couple amino acids to the growing peptide chain and to incorporate the Tz. We prepared a protected 1,4‐Triazole linker (see Supporting Information for synthetic details) as a dipeptide building block to assist with synthesizing Tz‐containing peptides. It was compatible with peptide synthesis on a commercial peptide synthesizer and enabled the synthesis of PCCs using a single automated program.

**Figure 4:**
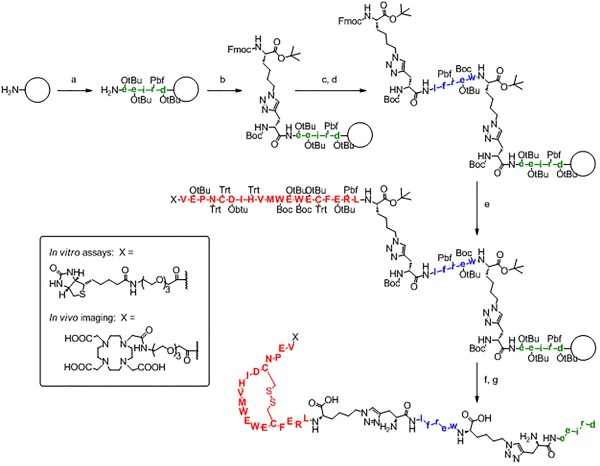
Solid‐phase synthesis of **Tri‐L_V_**. Amino acids are shown in one‐letter code, with l‐amino acids in uppercase and d‐amino acids in lowercase. *Reagents and conditions:* (A) standard Fmoc/HBTU chemistry; (B) 1,4‐Tz Linker (4 eq.), HATU (3.8 eq.), DIEA (10 eq.); (C) standard Fmoc/HBTU chemistry; (D) 1,4‐Tz Linker (4 eq.), HATU (3.8 eq.), DIEA (10 eq.); (E) standard Fmoc/HBTU chemistry; (F) TFA/H2O/TES (95/2.5/2.5) for 4 h, followed by ether precipitation; (G) intramolecular disulfide cyclization for 4–16 h in 0.05M ammonium acetate + 10% (v/v) DMSO at pH 7–8

Figure 4 illustrates the steps that were used in the synthesis of **Tri‐Lv** as an example. The tertiary ligand (eeird), 1,4‐Triazole linker, secondary ligand (lfrew), 1,4‐Triazole linker, and anchor (VEPNCDIHVMWEWECFERL) were coupled to the resin in series. Following global side chain deprotection and resin cleavage, the disulfide bridge was formed. The crude peptide was subsequently purified by HPLC on a C_18_ reversed‐phase column.

### 3.4 Anti‐protective antigen (PA) PCC biligand: stability studies

High thermal stability of a capture agent can enable the use of that ligand in demanding physical environments removed from refrigeration chains.^2^ The binding characteristics of antibodies and protein scaffolds to a specific antigen typically rely upon their secondary and/or tertiary structures in solution. When these folded structures are exposed to heat, the intramolecular forces and disulfide bonds that are required to hold the reagent in its native state are altered, resulting in an unfolded, inactive reagent that is not easily refolded. The denaturing phenomenon can be observed in the CD spectrum of a commercially available anti‐PA antibody heated to 90°C (Figure S15, Supporting Information). Short peptide reagents typically exhibit a weaker dependence upon secondary or tertiary structure for binding, and can, therefore, potentially display improved thermal stabilities in solution. The anti‐PA biligand (**Bi‐L_PA_**) follows this trend by exhibiting a random irregular structure in solution,^26^ based on circular dichroism measurements.^27^ The random coil structure of **Bi‐L_PA_** is unaffected after multiple rounds of heating up to 90°C.

**Figure 5:**
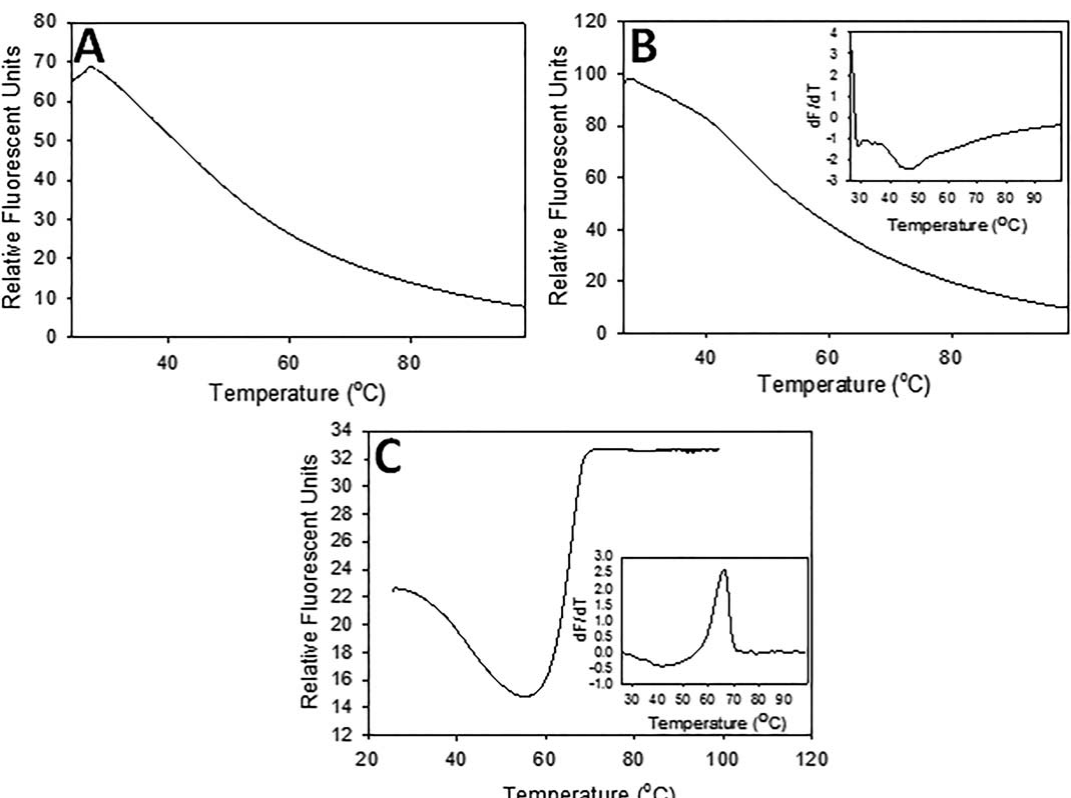
Thermal Shift Assay of (A) 1 mg/mL **Bi‐L_PA_**, (B) 15.5 mg/mL **Bi‐L_PA_**, and (C) anti‐PA monoclonal antibody. The insets of (B) and (C) correspond to the first derivative of the collected data

The unfolding of antibodies typically results in significant aggregation of the structures. Similar aggregation of peptides in solution is not uncommon,^28^ and in the case of bio‐detection, can greatly reduce the activity of the reagent. The onset of peptide aggregation is largely dependent upon amino acid sequence, with the content of hydrophobic residues typically leading to a higher probability of aggregation.^29^ **Bi‐L_PA_** aggregation was studied between 25°C and 99°C with a Thermal Shift Assay Kit (ProteoStat). The assay uses a fluorescent dye that detects protein aggregation resulting from thermal denaturation. At a concentration of 1 mg/mL, **Bi‐L_PA_** does not exhibit signs of aggregation (Figure 5A), whereas at a significantly higher concentration of 15.5 mg/mL there is only evidence of rapid biligand solvation occurring around 47°C, as seen in Figure 5B inset. As a comparison, the commercially available anti‐PA monoclonal antibody denatures and aggregates at 66°C (Figure 5C).

**Figure 6:**
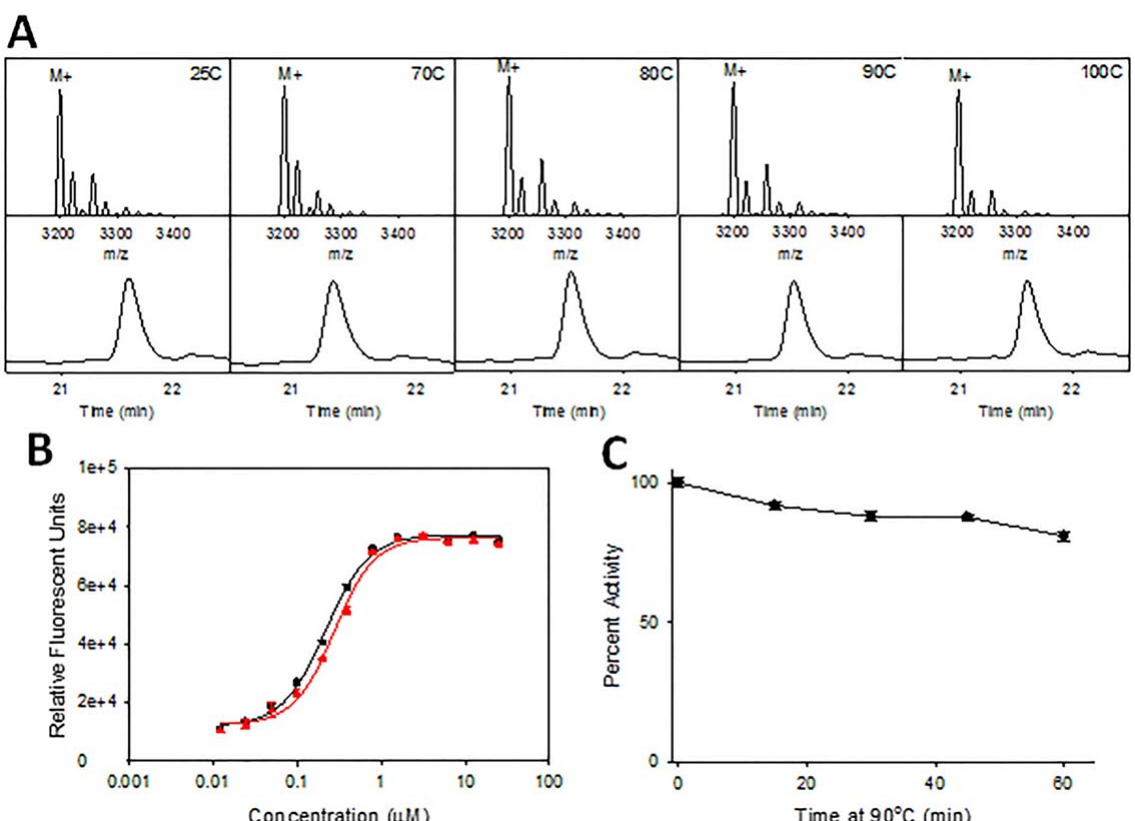
(A) Mass spectrum and HPLC trace of **Bi‐L_PA_** after 1 h heating at each respective temperature: 25°C, 70°C, 80°C, 90°C, and 100°C. (B) ELISA measurements of the **Bi‐L_PA_** before heating (black; Kd = 216 nM ± 7 nM) and after 1 h at 70°C (red; Kd = 283 nM ± 27 nM). (C) SPR activity assay of **Bi‐L_PA_** against PA at 90°C for 1 h

In terms of chemical stability, **Bi‐L_PA_** does not exhibit any fragmentation or significant structural change after heating for 1 h up to 100°C in solution, as determined by mass spectrometry (ms) and HPLC analysis (Figure 6A). The mass spectra reveal comparable patterning dominated by the M+ peak of the biligand, while the HPLC traces elute the sample at analogous times and with similar peak areas, along with no indication of an increase or introduction of decomposition products. In fact, **Bi‐L_PA_** must be heated up to ∼170°C in the solid state to observe the onset of a melting endotherm (Figure S16, Supporting Information), which is comparable to other peptide melting points.^30^ Not all products of chemical degradation will be detected via HPLC or ms analysis. For example, a deamidation reaction could rearrange asparagine to the inactive isoaspartate form,^31^ resulting in at most a 1 g/mol difference in molecular weight, with little to no change in the polarity of the specific amino acid side group. Thus, we further explored thermal stability via the impact of heating on immunoassays that employ the PCC.

#### 3.4.1 Thermal activity

We investigated the influence of temperature on **Bi‐L_PA_** in buffer solution through activity studies. A thermal challenge precedence was set during an antibody engineering study as part of the Defense Advanced Research Projects Agency (DARPA) Antibody Technology Program^32^ through accelerated lifetime testing. According to the DARPA standard, a highly effective reagent retains >85% activity after heating at 70°C for 1 h.^5^ As an initial test of the thermal effect on the activity of **Bi‐L_PA_**, we performed an ELISA before and after heating at 70°C for 1 h. As shown in Figure 6B, the *K*_D_ for unheated **Bi‐L_PA_** is 216 ± 7 n*M*, whereas the *K*_D_ after an hour is 283 nM ± 27 nM, suggesting insignificant reduction in K_D_ after the reagent is heated, surpassing the 70°C for 1 h precedence. The high efficacy of the reagent suggests that it is extremely competitive with antibody replacements reported in the literature that also demonstrate high thermal stability.^6^

We further investigated **Bi‐L_PA_** following heating under the more extreme condition of 90°C. By creating a linear calibration curve (Supporting Information, Figure S17) using relative R_max_ from the analyte injections of unheated biligand, we were able to plot responses of our heated samples and extrapolate initial sample concentrations based on the dilution factors used. The calculated concentrations were used to calculate the percent activity of each heated sample.^6^ The reagent retained 81% of its activity compared to unheated **Bi‐L_PA_** after 1 h of heating at 90°C, as shown in Figure 6C. The determined activity is ∼20% higher than the best known modified antibody at this high temperature and time scale,^5^ and to the best of our knowledge is the most thermally stable and functional biorecognition reagent known to date. The ability of **Bi‐L_PA_** and other reagents discovered through the PCC technology to retain activity at such high temperatures makes it an extremely effective reagent for detection of biothreats outside of a laboratory environment that can easily be incorporated into a wide range of detection devices.

#### 3.4.2 Isoaspartate content

The most significant contributor to a loss of activity of a non‐aggregating peptide with no secondary structure and no fragmentation upon heating is most likely caused by non‐enzymatic degradation. Commonly when a peptide is heated, the rate of intramolecular hydrolysis or deamidation reactions between amino acids greatly increases, yielding a mixture of isoaspartic acid (typically 70–85%) and aspartic acid.^33^ The rate of these reactions is sequence‐dependent and is most frequent when Asparagine, Aspartic acid, or Glutamine are present next to a Glycine in an oligopeptide sequence, though the Glutamine rearrangement occurs at a much slower rate.^34^ Based on the **Bi‐L_PA_** sequence (elfhn‐triazole‐YGLHPWWK**NA**PIGQR), the Asp‐Ala combination has the highest potential of rearranging to isoaspartate, which could diminish the activity of the PCC after heating.

The quantification of isoaspartate was performed on a solution of **Bi‐L_PA_** heated at 90°C for 1 h using an ISOQUANT Isoaspartate Detection Kit (Promega). Isoaspartate was present in the sample after 15 min of heating and increased in a linear fashion, as aliquots were tested every 15 min (Supporting Information, Figure S18). When compared to the total amount of biligand in the solution, there is only a small fraction of a percent of isoaspartate created per picomol of biligand after 1 h at 90°C, described in Table S1. While the amount of isoaspartate generated is low, it could help explain the loss of activity as determined by SPR at 90°C.

Understanding mechanisms for activity loss offers insight into the further design of the PCC peptide screening libraries, using the bottom‐up approach of this technology. One potential approach in reducing isoaspartate production is to substitute and screen different hydrophobic amino acid side chains in the Alanine position adjacent to Asparagine, providing an increase in steric hindrance, and reducing the opportunity for intramolecular cyclization. The Asparagine or Glutamine residues, themselves, could also potentially be substituted with other polar amino acid sidechains, preventing deamidation.

## 4 Conclusions

The capability of tailoring desired characteristics of capture agents including affinity/selectivity, size, chemical/biological stability, and temperature stability through the PCC screening process has the potential to provide highly effective synthetic antibody replacements for a range of *in vitro* and *in vivo* applications. The anti‐VEGF PCCs exhibit varying affinities and selectivities, dependent upon the degree of maturation, with a high degree of biological stability arising from the incorporation of d‐amino acids into the matured reagents. The *in vitro* performance of **Tetra‐L_V_** was closest to the Fab fragment of BVZ, the anti‐VEGF monoclonal antibody. **Tri‐L_V_** exhibited *in vivo* binding to VEGF and at an epitope shared with BVZ. We are exploring additional assays for guiding PCC development for *in vivo* performance, including ADME preclinical studies [absorption, distribution, metabolism, and excretion], imaging with PEGylated compositions, and drugging studies in a rodent tumor xenograft model.

The heating studies with an anti‐PA PCC show that the thermal stability of PCC agents is limited mainly by intramolecular reactions, leading to a slight loss in activity under prolonged exposure at extreme temperatures (90°C). The rate of isoaspartate production and other intramolecular interactions are mostly sequence‐dependent. This rate could likely be reduced by altering the amino acid composition of the initial screening library by completely removing the amino acids that exhibit deamidation and hydrolysis such as asparagine, aspartic acid, and glutamine. Additionally, substitution of these amino acids with unnatural analogs such as nitro or fluorinated side groups could also result in similar, if not better, performing properties. We are currently looking towards the use of cyclic peptides to provide even greater stability, since the rate of such intramolecular reactions is greatly reduced,^35^ and the cyclic peptides tend to yield higher affinities and selectivities from even fewer screening steps.^36^

## Supporting information

Supporting Information
