# Supplementary material for: Protein catalyzed capture agents with tailored performance for in vitro and in vivo applications
Source: Biopolymers. 2017 Mar 25;108(2):e22934. doi: 10.1002/bip.22934 (PMC6585716; doi:10.1002/bip.22934)
Supplement: Supplementary file 1 — Supporting Information [file BIP-108-na-s001.docx]

Supporting Information for

Protein Catalyzed Capture Agents with Tailored Performance for *In Vitro* and *In Vivo* Applications

Matthew B. Coppock^a^; Candice R. Warner^b^; Brandi Dorsey^c^; Joshua A. Orlicki^d^; Deborah A. Sarkes^a^; Bert T. Lai^e^; Suresh M. Pitram^e^; Rosemary D. Rohde^e^; Jacquie Malette^e^; Jeré A. Wilson^e^; Paul Kearney^f^; Kenneth C. Fang^f^; Scott M. Law^f^; Sherri L. Candelario^f^; Blake Farrow^g^; Amethist S. Finch^a^; Heather D. Agnew^e^; James R. Heath^g^; Dimitra N. Stratis-Cullum*^a^

*^a^ Sensors and Electron Devices Directorate, U.S. Army Research Laboratory, Adelphi, MD USA 20783; ^b^ Excet, Springfield, VA 22151 supporting USA Edgewood Chemical Biological Center, Aberdeen Proving Ground, MD USA 21010, ^c^ Federal Staffing Resources, 2200 Somerville Road, Suite 300, Annapolis, MD supporting U.S. Army Research Laboratory, Adelphi, MD USA 20783, ^d^ Weapons and Materials Research Directorate, U.S. Army Research Laboratory, Aberdeen Proving Ground, MD USA 21005, ^e^ Indi Molecular, 6162 Bristol Parkway, Culver City, CA 90230, ^f^ Integrated Diagnostics, Seattle, WA 98109, ^g^ Division of Chemistry and Chemical Engineering, California Institute of Technology, 1200 East California Boulevard, Pasadena, CA USA 91125*

Keywords: PCC Agent, Synthetic Antibody, Thermal Stability, Peptide, Protective Antigen, VEGF, Biological Stability

**METHODS**

**Synthesis of a 1,4-Triazole Linked Dipeptide (Tz4):**

Copper-catalyzed azide/alkyne cycloaddition (CuAAC)^1,2^ between a fully protected alkyne-containing amino acid and a fully protected azide-containing amino acid provided a protected 1,4-triazole linked dipeptide (Supporting Information, Scheme S1). Coupling conditions of this Tz4 linker on the CEM Liberty 1 microwave peptide synthesizer were modified to include 4 equiv of Fmoc-amino acid, 4 equiv of O-(7-azabenzotriazol-1-yl)-*N,N,N’,N’*-tetramethyluronium hexafluorophosphate (HATU), and 10 equiv of *N,N*-Diisopropylethylamine (DIEA). Deprotection of the Fmoc group required 20% (v/v) piperidine/NMP, followed by wash with NMP.

**Supporting Scheme S1.** (A) Synthesis of Fmoc-(L)-azidolysine t-butyl ester. (B) Copper catalyzed azide/alkyne cycloaddition (CuAAC) between a fully protected alkyne-containing amino acid and a fully protected azide-containing amino acid to provide a protected 1,4-triazole linked dipeptide.

1.
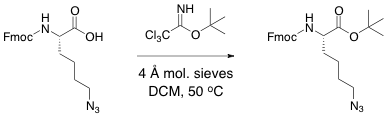

2.
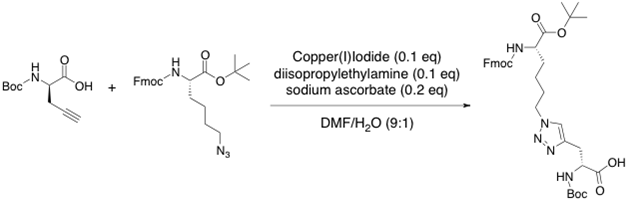


Synthesis of Fmoc-(L)-azidolysine t-butyl ester. Fmoc-(L)-azidolysine (2.23 mg, 5.65 mmol) was dissolved in dichloromethane (28 mL). To this solution was added 4-Å molecular sieves, followed by t-butyl-2,2,2-trichloroacetimidate (1.52 mL, 8.49 mmol). The reaction mixture was heated to 50 °C and stirred for 20 min, then an additional 1.52 mL (8.49 mmol) of t-butyl-2,2,2-trichloroacetimidate was added. The reaction was stirred at 50 °C for 16 h. The heterogeneous solution was cooled to 0 °C, then filtered to remove the sieves and white precipitate. Cold dichloromethane was used to wash the solid. The resulting solution was washed with saturated sodium bicarbonate and brine. The organic layer was dried over magnesium sulfate and concentrated. Flash chromatography (2% MeOH/DCM) gave the desired t-butyl ester derivative 1.21 g (2.69 mmol, 48% yield) as an oil.

CuAAC Procedure for 1,4-Tz Linker. Fmoc-(L)-Lys(N_3_)-Ot-Bu (950 mg, 2.1 mmol) and Boc-(D)-propargylglycine (451 mg, 2.1 mmol) were dissolved in a 9:1 mixture of DMF/H_2_O (7 mL). Copper(I) iodide (42 mg, 0.22 mmol) was added, followed by diisopropylethylamine (36 μL, 0.22 mmol). Sodium ascorbate (87 mg, 0.44 mmol) was dissolved in H_2_O (0.5 mL) and this aqueous solution was added to the reaction mixture. The reaction was stirred for 16 h. TLC (10% MeOH in DCM) indicated the presence of 1,4-triazole product and no propargylglycine starting material. The reaction mixture was diluted with EtOAC (35 mL) and sat. aq. NaHCO_3_ (25 mL). The aqueous layer was extracted with EtOAC (3 x 10 mL). The combined organic layers were washed with 0.1 M ammonium citrate (20 mL), followed by brine (10 mL), then dried over MgSO_4_, filtered and concentrated to give an oily solid. Purification by flash chromatography (5% to 10% MeOH in DCM) provided a white solid residue (1.3 g, 1.9 mmol, 93% yield).

**Maturation of an anti-VEGF peptide reagent:**

The phage display-derived, literature-based anti-VEGF cyclic peptide^3^ was synthesized with a pendant azide at the C-terminus to provide the **Anchor_V_** X-VEPNCDIHVMWEWECFERL-Az4 [where Az4 = L-azidolysine, X = biotin-PEG_3_ linker, and underlined = cyclized]. MALDI-MS (m/z): calcd. for C_139_H_203_N_35_O_39_S_4_ (M+) 3114.4; found 3114.5. Intramolecular disulfide cyclization was run over 4-16 h in 0.05 M ammonium acetate + 10% (v/v) DMSO at pH 7-8 (adjusted accordingly with 5% (w/v) aq. ammonium carbonate).

*In situ* click chemistry was used to screen the **Anchor_V_** against VEGF165 and an OBOC library of D-peptide 5-mers presenting the complementary alkyne (D-Pra = D-propargylglycine) using a pre-published method.^4^ The only modification to the screening process in comparison to the already-published method was the absence of the human serum anti-screen. Sequences were identified from bead hits and interrogated in 2 manners. First, sequence similarities were identified by visual inspection (Supporting Information, Figure S1). Second, the hit sequences were analyzed via a peptide analysis algorithm, Cluster Ligand v1.0, developed by Integrated Diagnostics (Indi), Seattle, WA. The algorithm uses principal component analysis to group peptides based on hydrophobicity and sequence homology, and graphs them on a multidimensional sequence map (Supporting Information, Figure S2). Different clusters are suggested to be indicative of targeting different protein epitope regions. Emphasis was placed on sequences at the periphery of the distribution, as these represent peptides with more distinct physicochemical properties. One peptide from each circled cluster was synthesized in bulk and tested for binding to VEGF165. Based on these combined approaches, 3 biligand candidates were selected and pursued (Biligand 1, Biligand 2, Biligand 3). These biligand candidates were synthesized and characterized by ELISA and immunoprecipitation studies (Supporting Information, Figures S3 and S4, respectively), as previously described.^4^ X-VEPNCDIHVMWEWECFERL-Tz4-rplir (Biligand 1). For X = acetyl, MALDI-MS (m/z): calcd. for C_151_H_226_N_44_O_39_S_3_ (M+) 3375.6; found 3375.8. For X = biotin-PEG_3_, MALDI-MS (m/z): calcd. for C_173_H_264_N_48_O_45_S_4_ (M+) 3861.9; found 3861.4. X-VEPNCDIHVMWEWECFERL-Tz4-lfrew (Biligand 2). For X = acetyl, MALDI-MS (m/z): calcd. for C_159_H_222_N_42_O_41_S_3_ (M^+^) 3471.6; found (M+H) 3473.3. For X = biotin-PEG_3_, MALDI-MS (m/z): calcd. for C_181_H_260_N_46_O_47_S_4_ (M^+^) 3957.8; found 3957.1. X-VEPNCDIHVMWEWECFERL-Tz4-fsrkte (Biligand 3). For X = acetyl, MALDI-MS (m/z): calcd. for C_155_H_225_N_43_O_44_S_3_ (M^+^) 3488.6; found 3489.9. For X = biotin-PEG_3_, MALDI-MS (m/z): calcd. for C_177_H_263_N_47_O_50_S_4_ (M^+^) 3974.8; found 3975.1.

Biligand 2 (**Bi-L_V_**) was taken into a subsequent *in situ* click chemistry screen against the same comprehensive library in the presence of VEGF165. Again, hit beads were isolated, and their sequences were processed for sequence similarities and informatics clustering (Supporting Information, Figures S5 and S6). Triligand candidates were selected, synthesized, and characterized by ELISA, immunoprecipitation, and inhibition assays (Supporting Information, Figures S7, S8, and S9). X-VEPNCDIHVMWEWECFERL-Tz4-lfrew-Tz4-frsvn (Triligand 1). For X = acetyl, MALDI-MS (m/z): calcd. for C_197_H_280_N_56_O_51_S_3_ (M^+^) 4343.0; found (M+Na) 4368.0. For X = biotin-PEG_3_, MALDI-MS (m/z): calcd. for C_219_H_318_N_60_O_57_S_4_ (M^+^) 4828.3; found 4830.6. X-VEPNCDIHVMWEWECFERL-Tz4-lfrew-Tz4-eeird (Triligand 2). For X = acetyl, MALDI-MS (m/z): calcd. for C_196_H_281_N_55_O_55_S_3_ (M^+^) 4382.0; found (M+H) 4383.3. For X = biotin-PEG_3_, MALDI-MS (m/z): calcd. for C_218_H_319_N_59_O_61_S_4_ (M^+^) 4867.3; found 4870.6. X-VEPNCDIHVMWEWECFERL-Tz4-lfrew-Tz4-hthwl (Triligand 3). For X = acetyl, MALDI-MS (m/z): calcd. for C_203_H_281_N_57_O_50_S_3_ (M^+^) 4413.0; found (M+H) 4415.2. X-VEPNCDIHVMWEWECFERL-Tz4-lfrew-Tz4-ewsrw (Triligand 4). For X = acetyl, MALDI-MS (m/z): calcd. for C_206_H_283_N_57_O_52_S_3_ (M^+^) 4483.0; found (M+H) 4484.6. For X = biotin-PEG_3_, MALDI-MS (m/z): calcd. for C_228_H_321_N_61_O_58_S_4_ (M^+^) 4969.3; found (M+K) 5008.0.

Triligand 2 (**Tri-L_V_**) was taken into a final *in situ* click chemistry screen against the same comprehensive library in the presence of VEGF165. Again, hit beads were isolated, and their sequences were processed for sequence similarities and informatics clustering (Supporting Information, Figures S10 and S11). Tetraligand candidates were selected, synthesized, and characterized by ELISA and immunoprecipitation (Supporting Information, Figures S12 and S13). X-VEPNCDIHVMWEWECFERL-Tz4-lfrew-Tz4-eeird-Tz4-yrpfw (Tetraligand 1). For X = biotin-PEG_3_, MALDI-MS (m/z): calcd. for C_263_H_372_N_72_O_69_S_4_ (M^+^) 5770.7; found 5773.3. X-VEPNCDIHVMWEWECFERL-Tz4-lfrew-Tz4-eeird-Tz4-qfkyr (Tetraligand 2). For X = biotin-PEG_3_, MALDI-MS (m/z): calcd. for C_258_H_375_N_73_O_70_S_4_ (M^+^) 5743.7; found 5745.7. Tetraligand 2 (**Tetra-L_V_**) was prioritized as the leading tetraligand against VEGF.

**Peptide Synthesis:**

Bulk synthesis of PCC candidates was carried out on 2-chlorotrityl chloride (CTC) resin (1.04 mmol/g) using microwave-assisted Fmoc-based solid-phase peptide synthesis (SPPS). The first amino acid was attached to the resin following the vendor’s protocol. The resin was transferred to the CEM Liberty 1 for the coupling of the remaining amino acids. Each amino acid coupling reaction incorporated 4 equiv of Fmoc-amino acid, 4 equiv of O-Benzotriazole-*N,N,N’,N’*-tetramethyl-uronium-hexafluoro-phosphate (HBTU), and 10 equiv of DIEA. Deprotection of the Fmoc group required 20% (v/v) piperidine/NMP, followed by wash with NMP. Intramolecular disulfide cyclization between cysteine residues was performed as described above. The crude peptides were precipitated with cold diethyl ether and subsequently purified by high-performance liquid chromatography on a C_18_ reversed-phase semi-preparative column (Phenomenex Luna, 5 µm, 250 × 10 mm). The purified peptides were utilized for screening, *in vitro*, and *in vivo* assays.

***In Vitro* Assays**

**VEGF ELISA.** Nunc MaxiSorp™ 96-well plates were coated with 2 µg/mL VEGF165 in PBS (pH 7.4) for 2 h. After washing wells with PBS (3 ×), the plate was incubated with blocking buffer (5% (w/v) milk in TBS (25 mM Tris, 150 mM NaCl, pH 7.25) containing 0.1% (v/v) Triton X-100) for 2 h. The plate was then washed with 1% (w/v) milk in TBS containing 0.1% (v/v) Triton X-100 (3 ×). Serial dilutions of biotinylated anti-VEGF PCC agents were treated to the appropriate wells for 2 h. After washing (3 ×), 0.1 µg/mL Streptavidin Poly-HRP conjugate (Pierce, 21140) in TBS/0.1% (v/v) Triton X-100 was incubated for 30 min. The plate was washed with TBS/0.1% (v/v) Triton X-100 (5 ×), followed by TBS (5 ×). QuantaRed™ Enhanced Chemifluorescent HRP Substrate was used to develop the microwells. Using an excitation wavelength of 535 nm, fluorescent emission at 595 nm was recorded. EC_50_ values were determined by fitting the titration curves with a 4-parameter regression model.

***In Vitro* Inhibition of VEGF Binding to VEGFR2.** To measure the ability of PCC agents to inhibit human VEGF165 binding to its cognate receptor, ELISA plates were coated with 10 µg/mL rabbit F(abꞌ)_2_ to human IgG Fc (Jackson ImmunoResearch, 309-006-008) in 50 mM carbonate buffer, pH 9.6, at 25 °C for 2 h and blocked overnight at 4 °C with the blocking buffer. Recombinant human VEGFR2 (KDR), Fc chimera (10 µg/mL; R&D Systems, 357-KD) in TBS containing 0.1% (v/v) Triton X-100 was incubated on the plate for 1 h at 25 °C. Three-fold serial dilutions of PCC agent or bevacizumab Fab (BVZ Fab) were incubated with 10 nM biotinylated VEGF165 in the washing buffer for 2 h separately. The solutions were then transferred to the ELISA plates and incubated for 5 min. The plate was washed with the washing buffer, followed by TBS/0.1% (v/v) Triton X-100 (3 ×). Bound biotinylated VEGF165 was detected using 0.2 µg/mL horseradish peroxidase-labeled streptavidin (Abcam, ab7403) prepared in TBS containing 0.1% (v/v) Triton X-100. The development and subsequent fitting of titration curve were performed following the protocols listed above. Concentrations of PCC agents corresponding to the midpoint absorbance of the titration curve were calculated and used as the IC_50_ values.

**Immunoprecipitation of VEGF.** Biotinylated PCC agents (400 nM; 0.1% DMSO, v/v) were incubated with 1 µg/mL VEGF165 in TBS or 25% (v/v) human AB male serum (Omega Scientific, HS-20) at 4 ^o^C overnight. A vehicle-only control (0.1% DMSO, v/v) accompanied each sample. BSA-blocked Dynabeads® M-280 Streptavidin (Invitrogen, 112-05D) were added to capture the proteins under rotation at 4 ^o^C for 4 h (100 µL of 50% slurry per pull-down condition). Beads were separated from the serum or buffer matrix by application of the DynaMag™-Spin magnet, and captured proteins were eluted from the beads in 30 µL of reducing Laemmli buffer. Eluted samples were subjected to 12% SDS-PAGE separation at 200 V for 30 min in 1x TGS (25 mM Tris, 192 mM Glycine, 0.1% SDS (w/v), pH 8.3). Samples were subsequently electrophoretically transferred to a nitrocellulose membrane in 25 mM Tris, 192 mM Glycine, pH 8.3, containing 20% (v/v) methanol at 100 V for 40 min.

Following transfer, the nitrocellulose membrane was blocked at 4 ^o^C for 2 h in 5% (w/v) milk in TBS. The membrane was then washed with TBS (3 ×), and incubated with 1 µg/mL mouse anti-human VEGF165 antibody [6B7] (Abcam, ab69479) in 0.5% (w/v) milk in TBS at 4 ^o^C overnight. After washing with TBS containing 0.02% Tween20 (v/v) (5 ×), 0.2 µg/mL HRP-conjugated goat polyclonal secondary antibody to mouse IgG (H + L) (Abcam, ab6789) in 0.5% (w/v) milk in TBS was added to the membrane (4 °C, 1 h incubation). After washing with TBS/0.02% Tween20 (v/v) (5 ×), followed by TBS (5 ×), the membrane was developed with SuperSignal® West Pico Chemiluminescent Substrate (Pierce, 34087) and exposed to HyBlot CL AR film.

**Circular Dichroism (CD) of Anti-PA.** CD measurements were performed on a Jasco J-815 CD spectrometer. 1 µM of anti-PA Mab in 10 mM potassium phosphate buffer pH 8.0 was heated from 20 ^o^C to 95 ^o^C, with a temperature gradient of 10 ^o^C/h and a 10-sec delay time. A spectrum was accumulated 3 times every 10 ^o^C, with continuous scanning at a speed of 20 nm/min between 260 nm and 185 nm. The data were smoothed using the negative exponential algorithm in SigmaPlot 12.

**Differential Scanning Calorimetry of Anti-PA.** The DSC experiment was performed on a TA Instruments Q1000 and the data was analyzed with Universal Analysis 2000 software. 0.6 mg of **Bi-L_PA_** was placed in a hermetically sealed aluminum pan. A heat-cool-heat cycle was surveyed, where the temperature was ramped from 40 ^o^C to 250 ^o^C at 10 ^o^C/min, lowered to 0 ^o^C at 20 ^o^C/min, and increased again to 250 ^o^C at 10 ^o^C/min.

**Isoaspartate Quantification Assay.** The quantification of isoaspartate resulting from the deamidation and/or hydrolysis of certain amino acids in the **Bi-L_PA_** was conducted with an ISOQUANT Isoaspartate Detection Kit (Promega). 500 µL of a 1 mg/mL sample of **Bi-L_PA_** was prepared in PBS, aliquoted in 100 µL volumes, and heated at 90 ^o^C for 15, 30, and 60 min. After each time point, the sample was immediately put on ice. Each aliquot was reacted in the master mix, as described in the included kit protocol in triplicate. 40 µL of the resulting reaction mixtures was injected into an Agilent 1200 series analytical HPLC instrument connected to a Synergi Hydro-RP HPLC column (Phenomenex). The mobile phase composition of MeOH and 50 mM potassium phosphate pH 6.2 was varied, as described in the included kit protocol. The area of the peaks corresponding to the S-adenosyl homocystein (SAH) product in each reaction sample was determined. A standard curve consisting of data points from the peak area of 5, 12.5, 25, 37.5, and 50 pmol of supplied SAH standard in nanopure water was used to determine the unknown isoaspartate amount in the biligand samples.

**Figure S1.** Hit sequences from the anti-VEGF PCC biligand screen. Red = positively charged side group, Green = aromatic side group, Yellow = polar side group, Blue = negatively charged side group, and White = neutral side group.


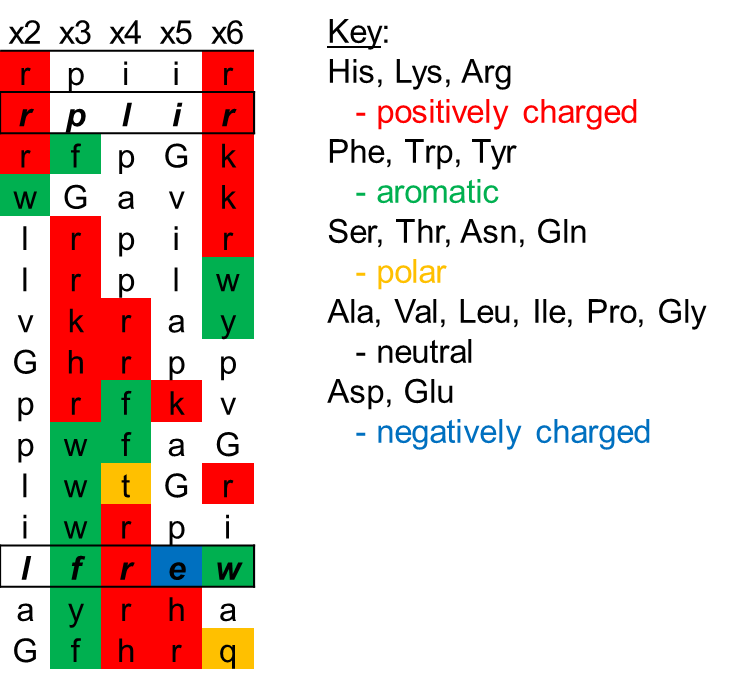


**Figure S2.** Informatic clustering analysis of anti-VEGF PCC secondary ligand candidates. Dark blue = 5-mer secondary ligand hits from biligand screen.


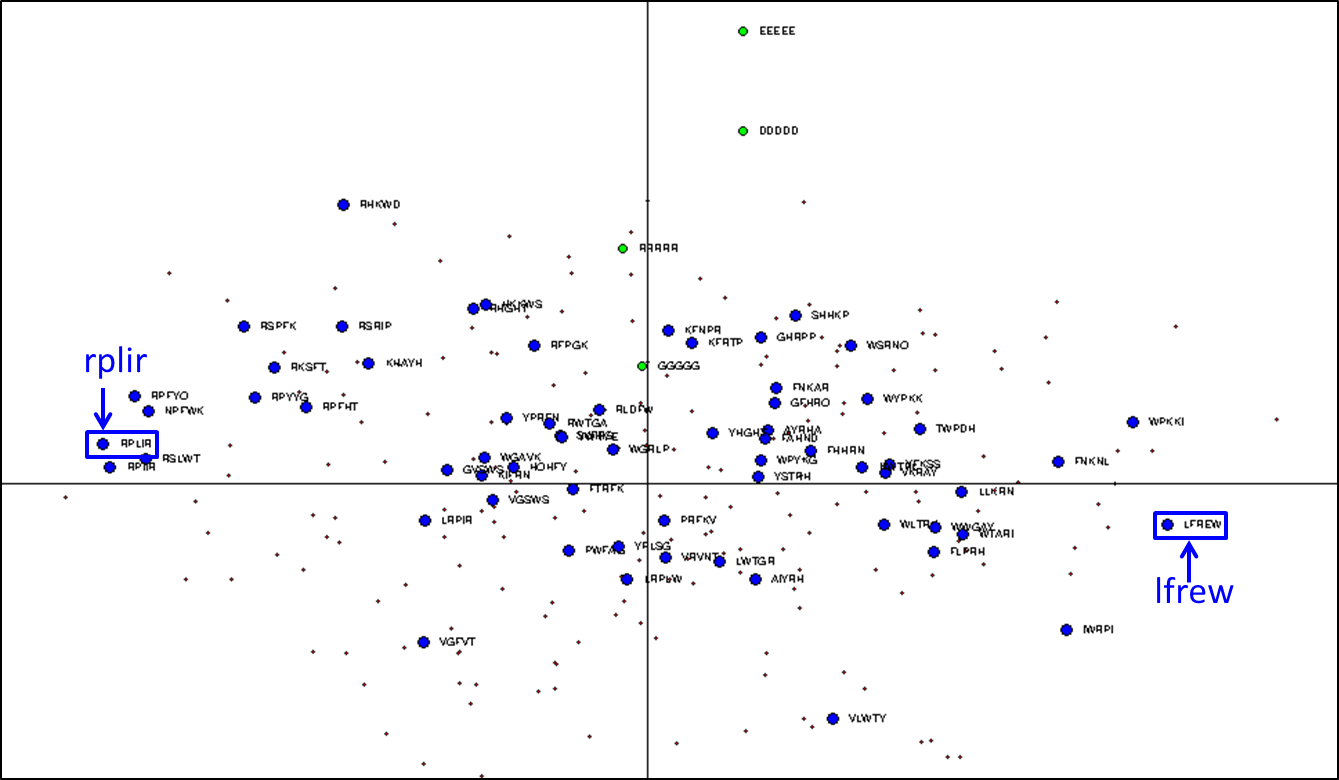


**Figure S3.** Anti-VEGF PCC biligand candidates vs. anti-VEGF anchor ELISA.

**Figure S4.** Immunoprecipitation of VEGF by anti-VEGF PCC biligand candidates vs. anti-VEGF anchor from buffer (P) and 25% human serum (S).

**
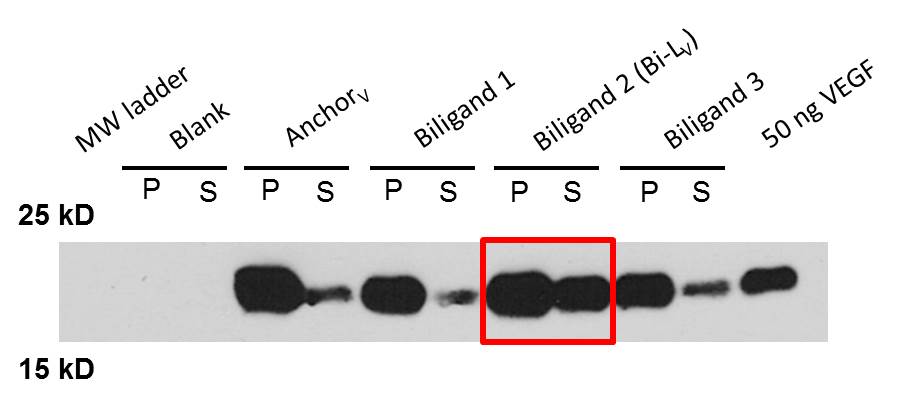
**

**Figure S5.** Hit sequences obtained from the anti-VEGF PCC triligand screen.


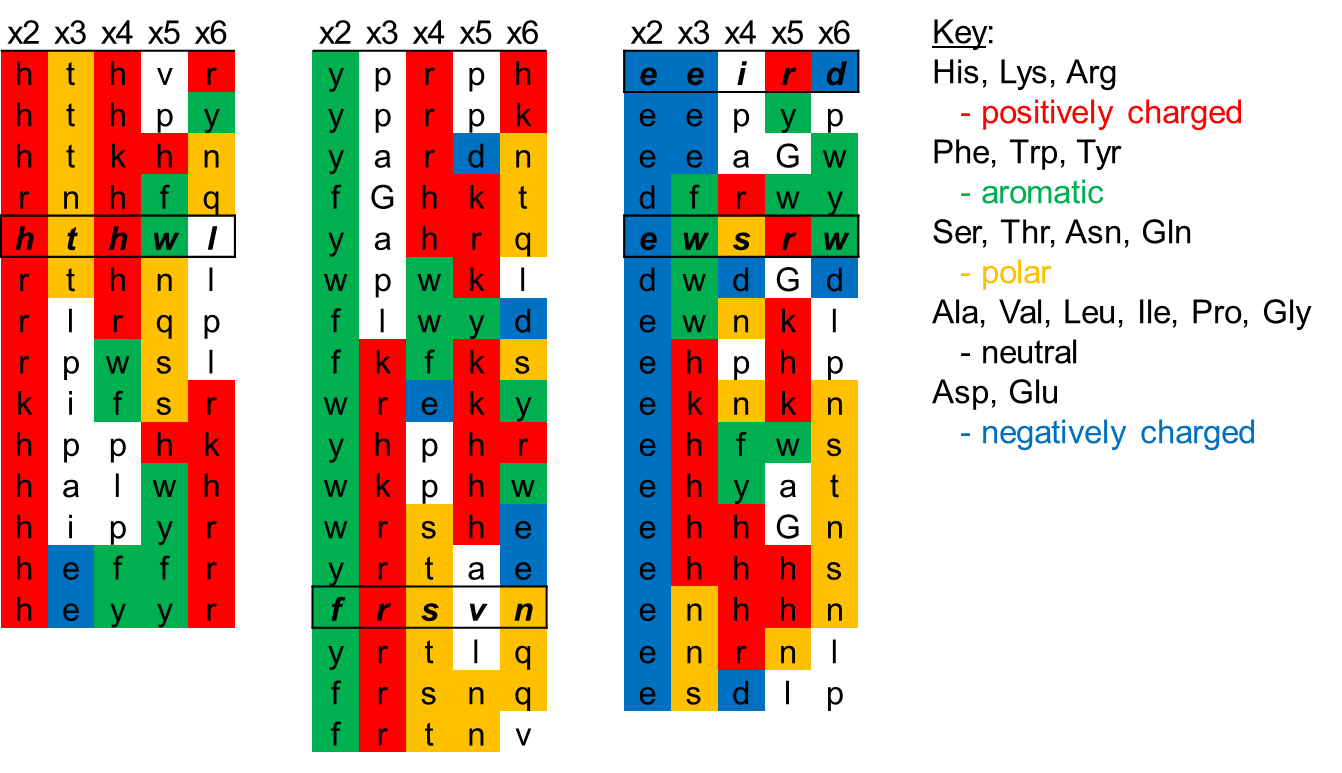


**Figure S6.** Informatic clustering analysis of anti-VEGF PCC tertiary ligand candidates. Dark blue = 5-mer tertiary ligand hits from triligand screen.


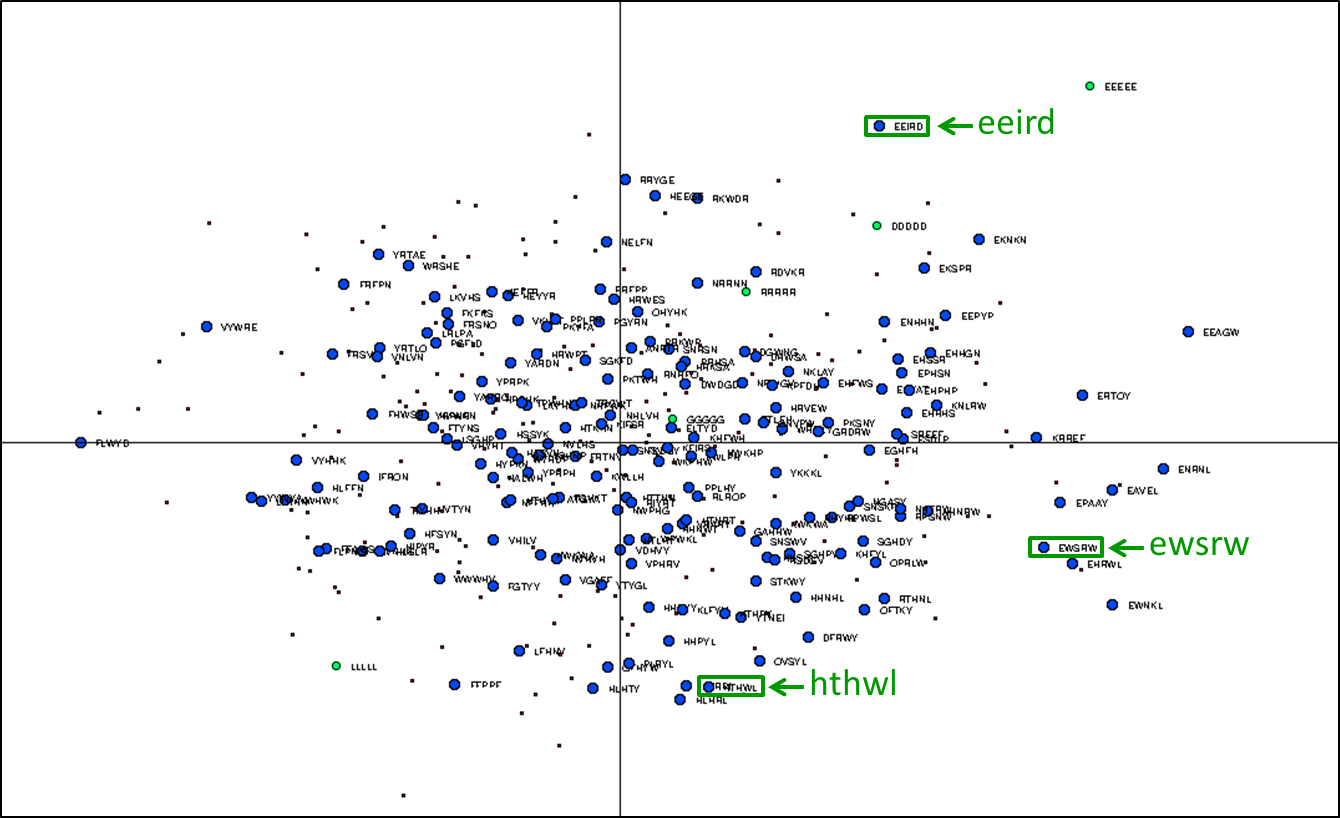


**Figure S7.** ELISA affinities of anti-VEGF PCC triligand candidates vs. **Anchor_V_**, Bevacizumab Fab (BVZ Fab), and Bevacizumab (BVZ).

**Figure S8.** Immunoprecipitation of VEGF by anti-VEGF PCC triligand candidates from buffer (P) and 25% human serum (S).

**
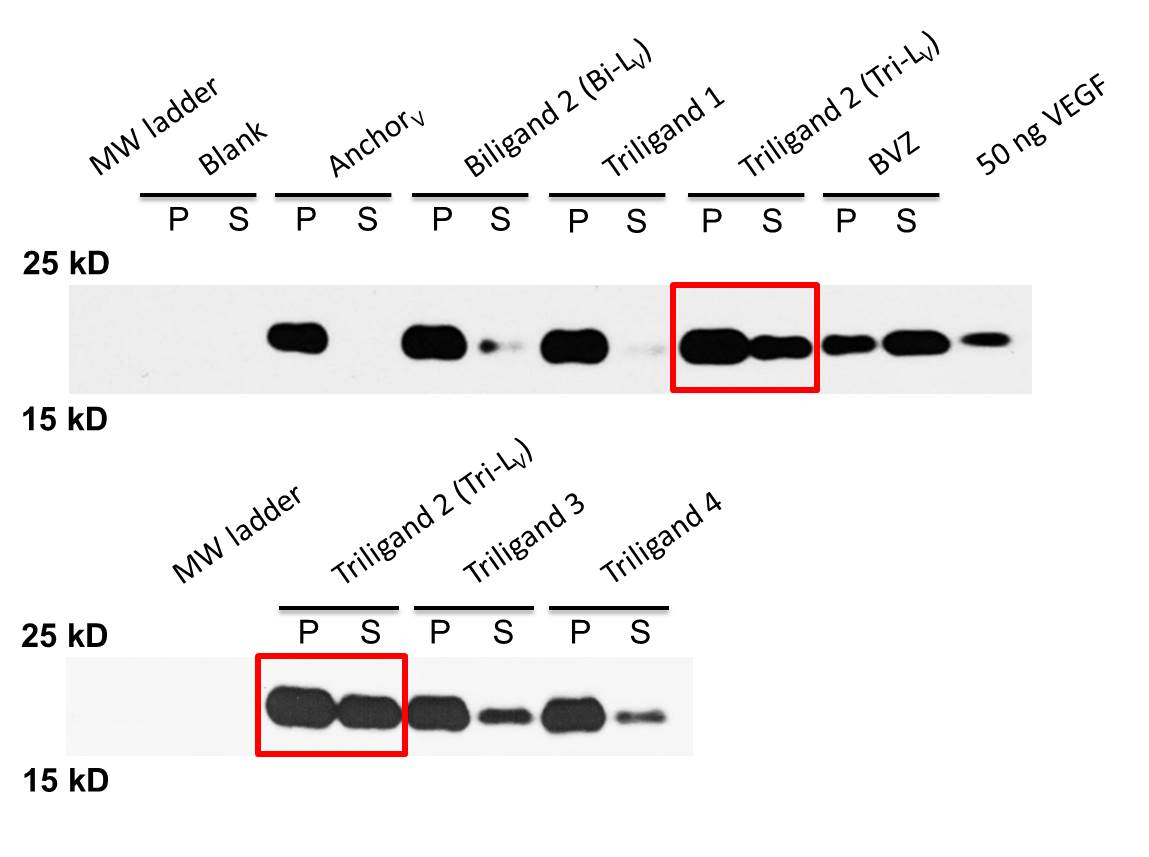
**

**Figure S9.** Inhibition of VEGF binding to VEGFR2 by anti-VEGF PCC triligand candidates. Receptor blocking activities were screened by measuring biotinylated VEGF165 binding to VEGFR2-coated wells in the presence of serial dilutions of PCC or BVZ Fab.

**Figure S10.** Hit sequences obtained from the anti-VEGF PCC tetraligand screen. Red = positively charged side group, Green = aromatic side group, Yellow = polar side group, Blue = negatively charged side group, and White = neutral side group.

**
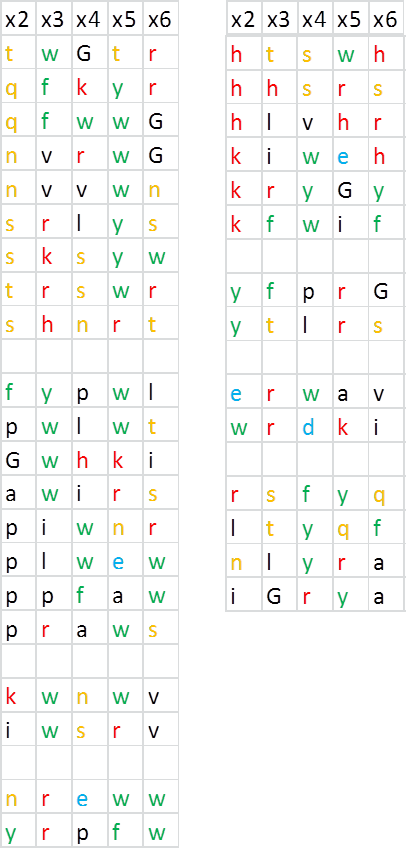
**

**Figure S11.** Informatic clustering analysis of anti-VEGF tetraligand candidates. Dark blue = 5-mer quaternary ligand hits from anti-VEGF tetraligand screen.

**
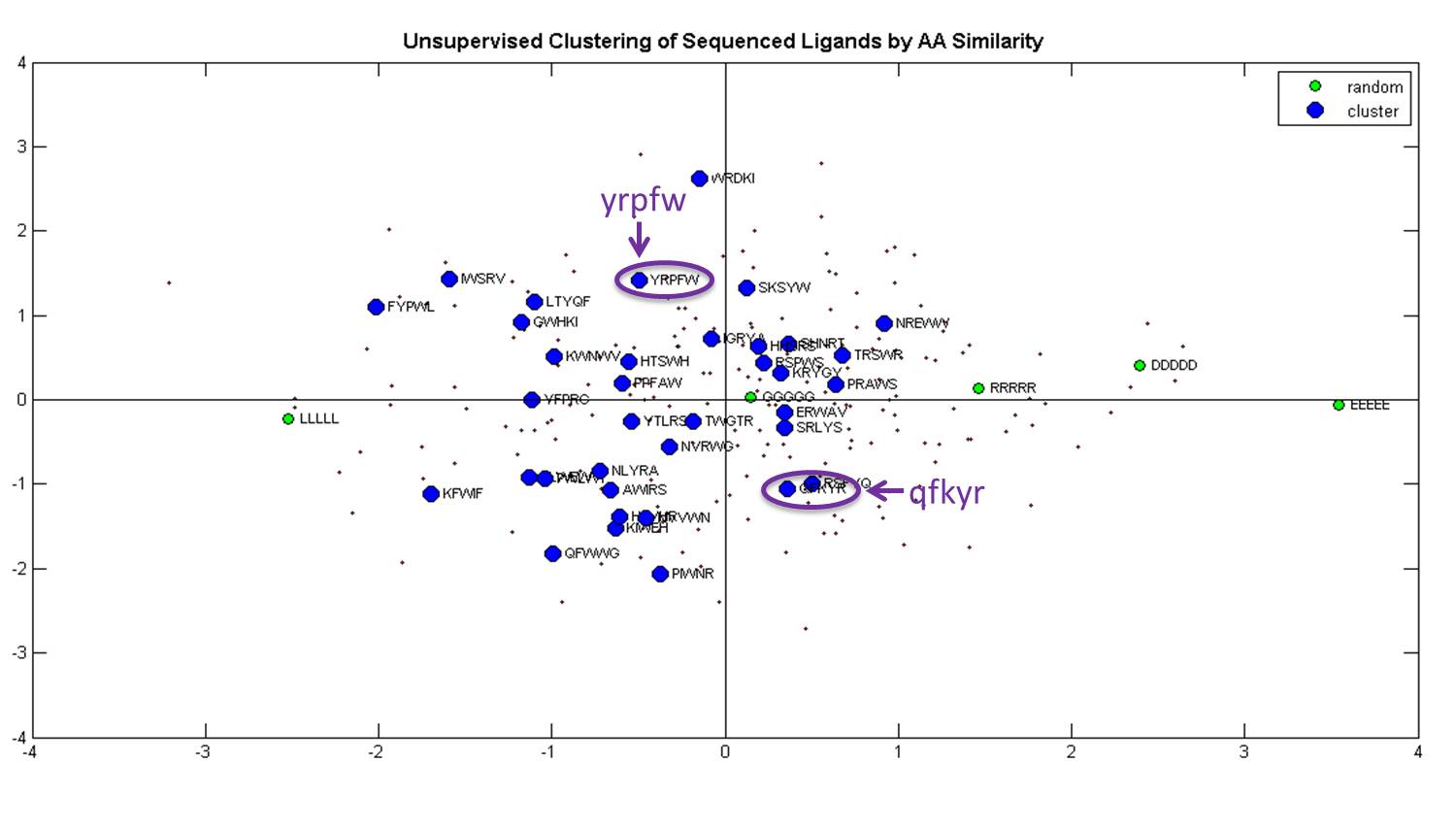
**

**Figure S12.** ELISA affinities of anti-VEGF PCC tetraligand candidates vs. the downselected anti-VEGF PCC triligand.

**Figure S13.** Anti-VEGF PCC tetraligand specificities evaluated by immunoprecipitation of VEGF from buffer (P) and 25% human serum (S).

**
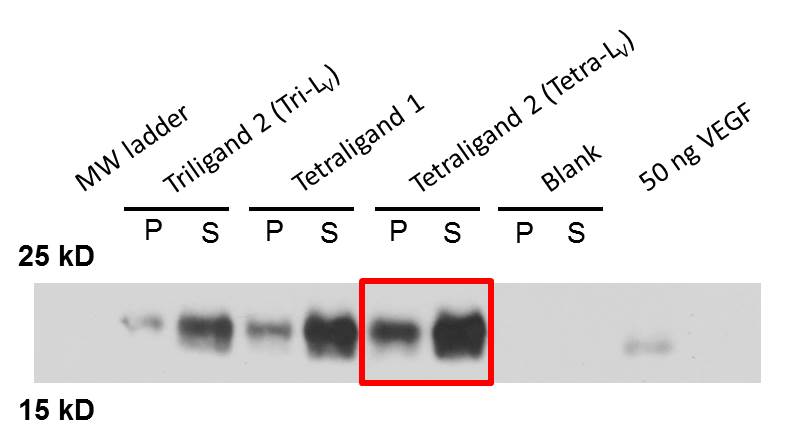
**

Figure S14. *In vivo* imaging of human VEGF in xenograft tumor. (A) *In vivo* microPET-CT images (coronal slices) of HT-29 tumor-bearing nude mice at 20 h after IP injection of ^64^Cu-DOTA-labeled Tri-L_V_. (B) For blocking human VEGF, unlabeled BVZ (1 mg) was administered IV 48 h prior to IP injection of ^64^Cu-DOTA-labeled Tri-L_V_. *In vivo* microPET-CT images collected at 20 h show a reduction of Tri-L_V_ in the tumor, suggesting that Tri-L_V_ and BVZ compete for binding to VEGF.

L, left kidney; R, right kidney; T, tumor. Images shown are representative of 4 mice per group.


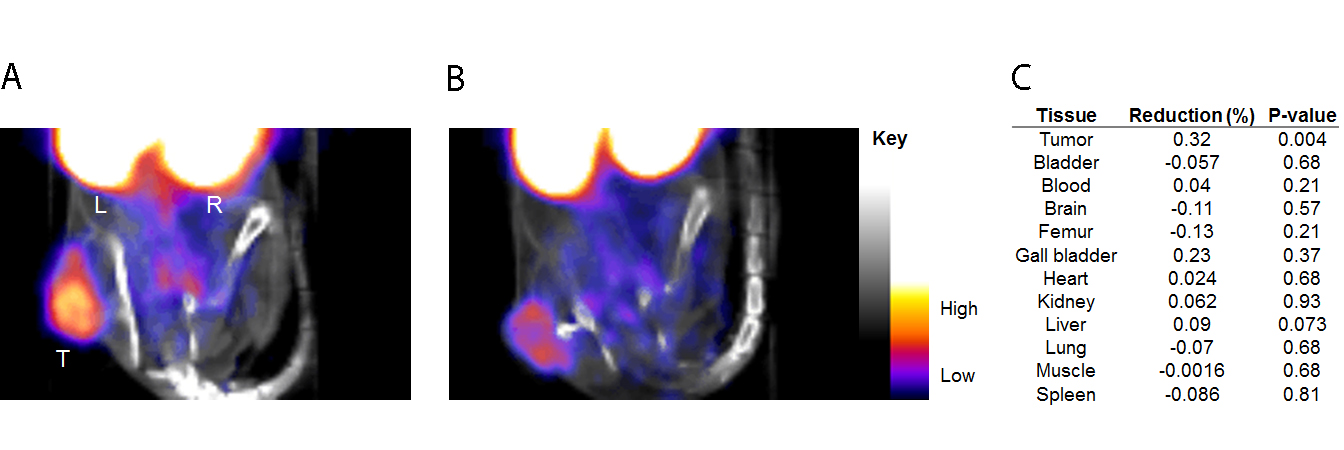


**Figure S15.** Circular Dichroism spectrum of the anti-Protective Antigen antibody at 20 ^o^C (black) and after heating at 90 ^o^C (red).

**Figure S16.** Differential scanning calorimetry trace for the **Bi-L_PA_**.

**Figure S17.** **Bi-L_PA_** SPR calibration curve with sample plots.

**Figure S18.** (A) Overlay of Isoaspartate Quantification Assay HPLC traces after heating **Bi-L_PA_** at 90 ^o^C for the specified durations. (B) Plot of HPLC peak areas, which are directly proportional to the amount of isoaspartate, against the sample heating time.

Table S1. Amount of isoaspartate in solution after heating at 90 ^o^ C for up to 60 min.


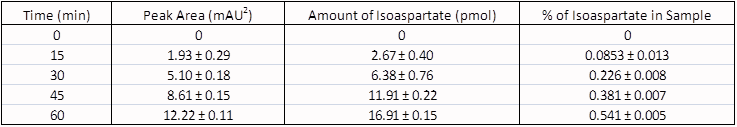


**Table S2.** General procedures, *in vivo* PK (following Hosten et al. ^5^).

| ***Assay*** | ***Source*** | ***Dose*** | ***Technique*** |
| --- | --- | --- | --- |
| PK in-life (mouse, IV, parallel sampling) | Male mice CD-1, weighing 20-30 g | 1 mg/kg | Tail vein injection/ blood collection |
| PK in-life (mouse, IP, parallel sampling) | Male mice CD-1, weighing 20-30 g | 5 mg/kg | Gastric gavage/ blood collection |

**Table S3.** Experimental conditions, *in vivo* PK.

| ***Assay*** | ***Sampling Time Points*** |
| --- | --- |
| PK in-life (mouse, IV, parallel sampling) | 3, 10, 30, 60, 120, 240, 360, 1440 min |
| PK in-life (mouse, IP, parallel sampling) | 10, 30, 60, 120, 240, 360, 480, 1440 min |

**Table S4.** Animal dosing design, *in vivo* PK (non-cannulated, non-fasted mice).

| ***Group*** | ***Experiment**** |
| --- | --- |
| 1 | **Bi-L_V_**, IV, n = 3 mice per time point (24 animals total) |
| 2 | **Bi-L_V_**, IP, n = 3 mice per time point (24 animals total) |
| 3 | **Tri-L_V_**, IV, n = 3 mice per time point (24 animals total) |
| 4 | **Tri-L_V_**, IP, n = 3 mice per time point (24 animals total) |
| 5 | Control animals (for drug-free blood), n = 6 mice |

Noncompartmental pharmacokinetic analysis^6^ was applied to the mean PCC agent plasma concentration data for mice. The following parameters were estimated whenever possible and are reported in Supporting Information, Table S5:

C_0_ Back extrapolated concentration at time 0.

C_max_ Maximum observed plasma concentration.

AUC_0-t_ Area under the concentration‑time curve from time 0 to the last measurable concentration

AUC_inf_ Area under the concentration-time curve from time 0 to infinity.

AUC% Ext Percent of the area under the curve extrapolated from the last measurable concentration to infinity.

T_1/2_ Elimination half-life.

CL Systemic plasma clearance.

T_max_ Time corresponding to maximum observed plasma concentration.

V_ss_ Volume of distribution at steady-state.

F% Relative bioavailability.

**Table S5.** Mean pharmacokinetic parameters for anti-VEGF PCC agents in mouse plasma following a single IV or IP dose.

| Compound | | Dose Route | | Dose  (mg/kg) | | AUC_0-t_ (hr*ng/mL) | AUC_inf_ (hr*ng/mL) | | AUC% Ext (%) | CL (mL/min/kg) | | F (%) | T_1/2_ (min) | C_max_ (ng/mL) | T_max_ (hr) | V_ss_ (L/kg) |
| --- | --- | --- | --- | --- | --- | --- | --- | --- | --- | --- | --- | --- | --- | --- | --- | --- |
| **Bi-L_V_** | IV | | 1 | | 1046 | | 1109 | 5.68 | | 15 | NA | | 7 | NA | NA | 0.160 |
| **Bi-L_V_** | IP | | 5 | | 2167 | | 2424 | 10.6 | | NA | 41 | | 154 | 3207 | 0.17 | NA |
| **Tri-L_V_** | IV | | 1 | | 6070 | | 6490 | 6.49 | | 2.57 | NA | | 36 | NA | NA | 0.0967 |
| **Tri-L_V_** | IP | | 5 | | 31500 | | 32200 | 2.04 | | NA | 99 | | NA | 19800 | 0.50 | NA |

NA Not applicable

**Table S6.** Mobile phases, HPLC column, and gradient program used for analyzing the human plasma stability of anti-VEGF PCC agents.


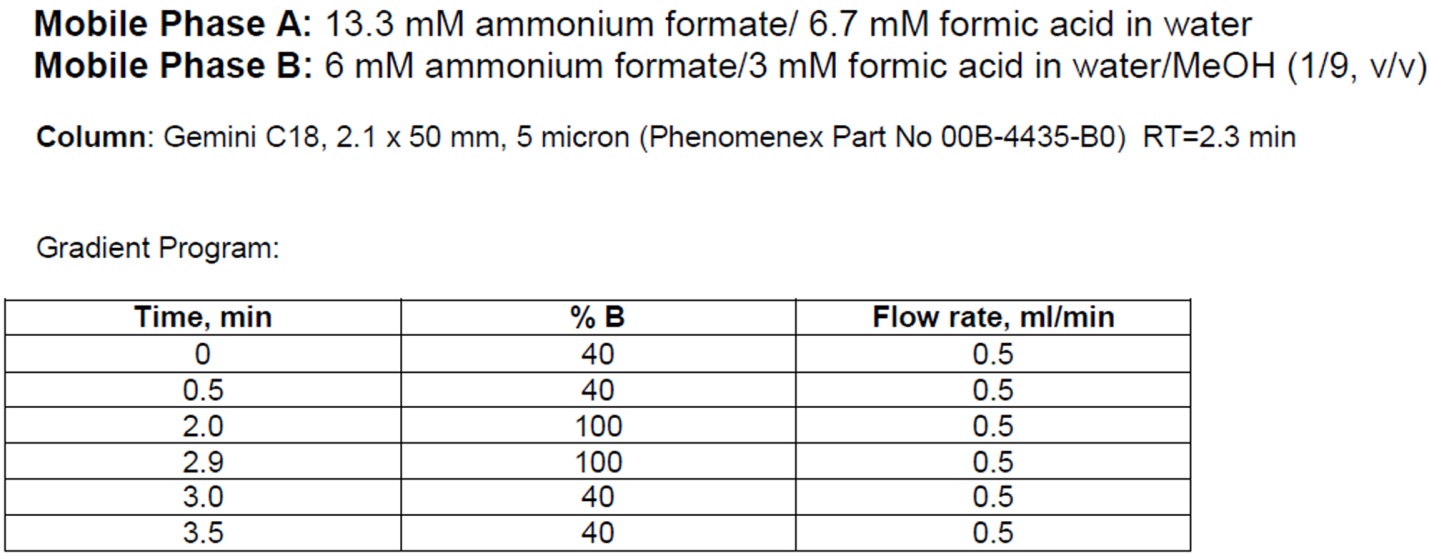


**REFERENCES**

1. Rostovtsev, V. V.; Green, L. G.; Fokin, V. V.; Sharpless, K. B., A Stepwise Huisgen Cycloaddition Process: Copper(I)-Catalyzed Regioselective “Ligation” of Azides and Terminal Alkynes. *Angew. Chem. Int. Ed.* **2002,** *41* (14), 2596-2599.

2. Tornøe, C. W.; Christensen, C.; Meldal, M., Peptidotriazoles on Solid Phase:  [1,2,3]-Triazoles by Regiospecific Copper(I)-Catalyzed 1,3-Dipolar Cycloadditions of Terminal Alkynes to Azides. *J. Org. Chem.* **2002,** *67* (9), 3057-3064.

3. Fairbrother, W. J.; Christinger, H. W.; Cochran, A. G.; Fuh, G.; Keenan, C. J.; Quan, C.; Shriver, S. K.; Tom, J. Y. K.; Wells, J. A.; Cunningham, B. C., Novel Peptides Selected to Bind Vascular Endothelial Growth Factor Target the Receptor-Binding Site. *Biochemistry* **1998,** *37* (51), 17754-17764.

4. Farrow, B.; Hong, S. A.; Romero, E. C.; Lai, B.; Coppock, M. B.; Deyle, K. M.; Finch, A. S.; Stratis-Cullum, D. N.; Agnew, H. D.; Yang, S.; Heath, J. R., A Chemically Synthesized Capture Agent Enables the Selective, Sensitive, and Robust Electrochemical Detection of Anthrax Protective Antigen. *ACS Nano* **2013,** *7* (10), 9452-9460.

5. Hosten, B.; Abbara, C.; Petit, B.; Dauvin, A.; Bourasset, F.; Farinotti, R.; Gonin, P.; Bonhomme-Faivre, L., Effect of Interleukin-2 Pretreatment on Paclitaxel Absorption and Tissue Disposition after Oral and Intravenous Administration in Mice. *Drug Metabolism and Disposition* **2008,** *36* (8), 1729-1735.

6. Gibaldi, M.; Perrier, D., *Pharmacokinetics*. Marcel Dekker: New York, New York, 1982.
